# Supplementary material for: Gut bacterial metabolite imidazole propionate potentiates Alzheimer’s disease pathology
Source: Nat Commun. 2026 Jun 26;17:8017. doi: 10.1038/s41467-026-74744-z (PMC13454616; doi:10.1038/s41467-026-74744-z)
Supplement: Supplementary file 2 [file 41467_2026_74744_MOESM1_ESM.pdf]

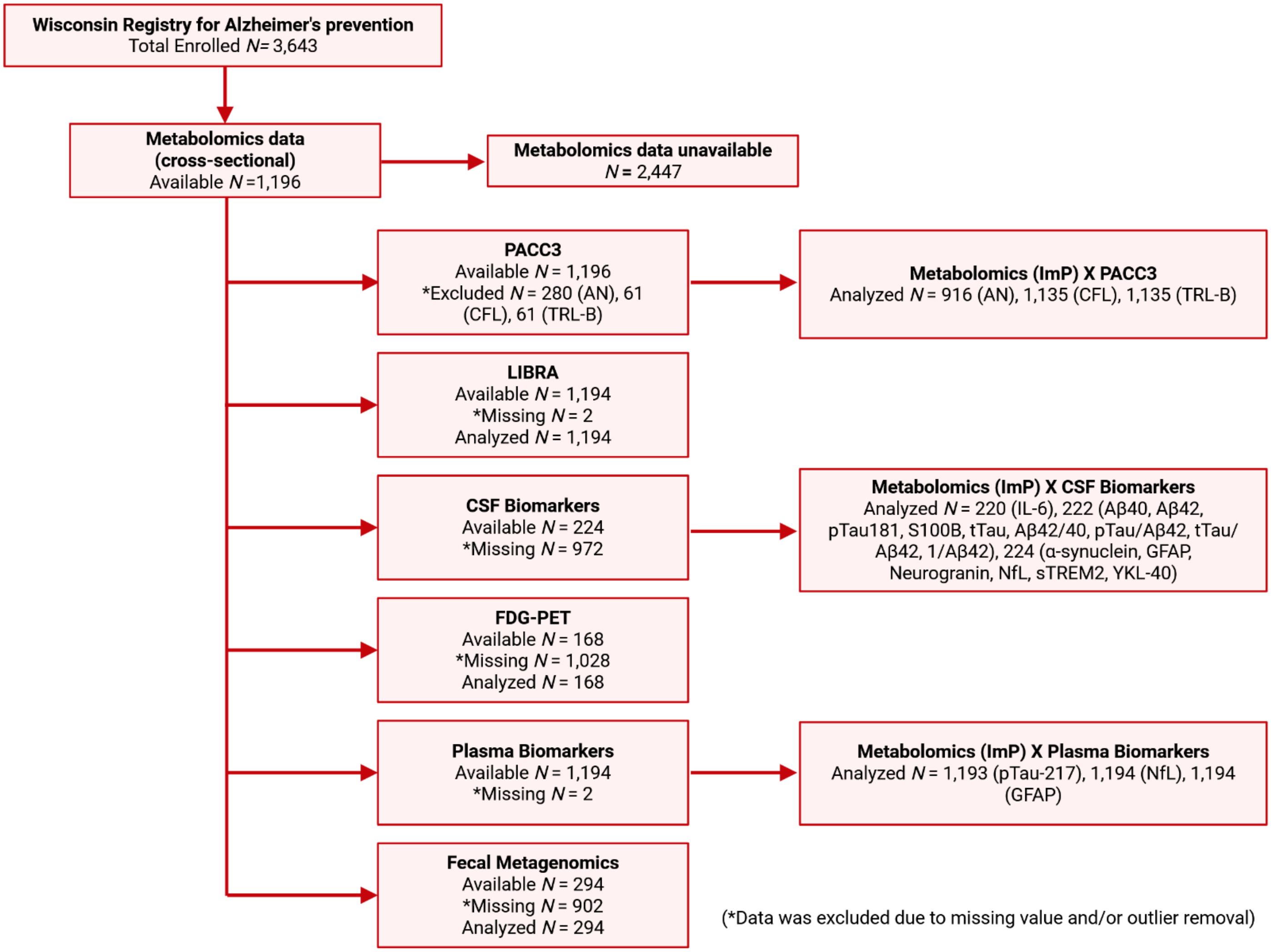

Supplementary Figure 1. CONSORT flow diagram summarizing demographic information for cross-sectional assesment of associations between ImP and ADRD phenotypes.

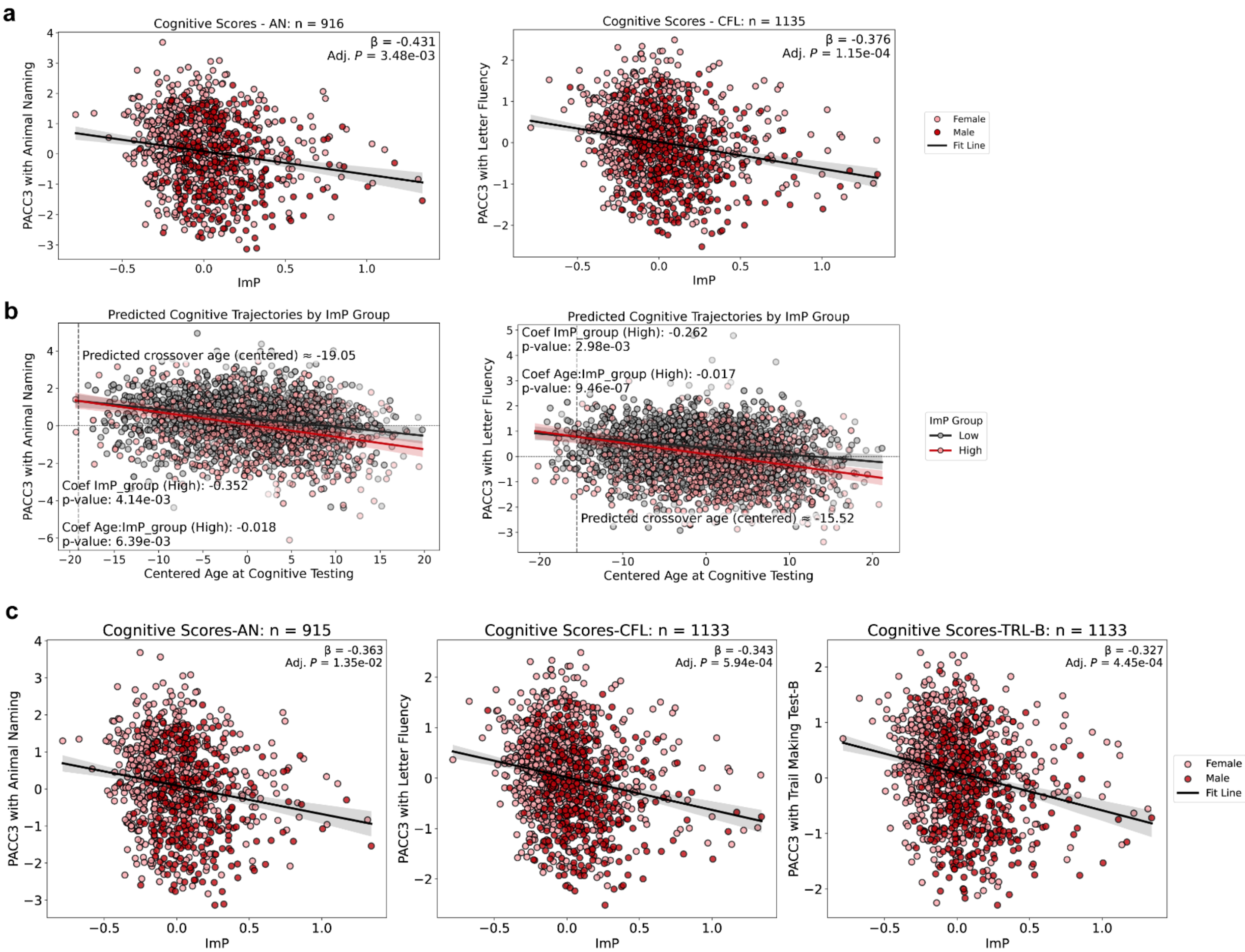

Supplementary Figure 2. Plasma ImP is associated with lowered mPACC3 cognitive scores. a, Plasma ImP is negatively associated with mPACC3-AN (*N* = 916, *P* = 3.48e−03, OLS regression, β [95% CI] for ImP = −0.431), and mPACC3-CFL (*N* = 1135, *P* = 1.15e−04, OLS regression, β [95% CI] for ImP = −0.376), sex is represented by pink (females) and red (males). **b,** Modeled cognitive trajectories over age for mPACC3 scores (AN and CFL), with the vertical dashed line indicates predicted crossover age and the horizontal dashed line denotes zero on the scaled y-axis, comparing top (red) and bottom (gray) ImP quartiles. **c,** Plasma ImP is associated with pre-clinical cognitive scores agnostic to comorbid LIBRA scores as a covariate, mPACC3-AN (*N* = 915, *P* = 1.35e−02, OLS regression, β [95% CI] for ImP = −0.363), mPACC3-CFL (*N* = 1133, *P* = 5.94e−04, OLS regression, β [95% CI] for ImP = −0.343), and mPACC3 TRL-B (*N* = 1196, *P* = 4.45e−04, OLS regression, β [95% CI] for ImP = −0.327), pink (females) and red (males).

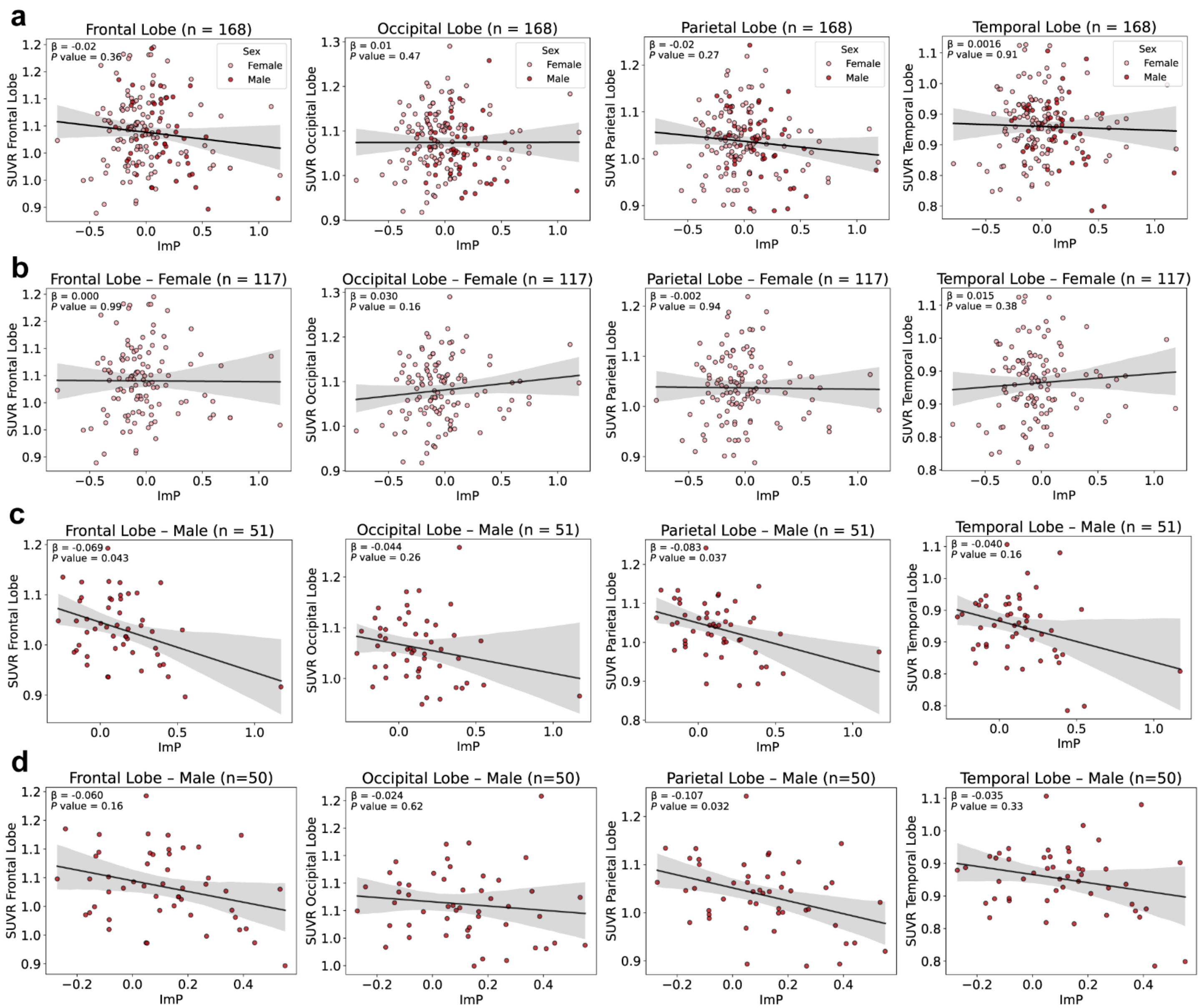

Supplementary Figure 3. Plasma ImP levels are not significantly associated with FDG SUVR. a, Plasma ImP levels are not significantly associated with FDG SUVR in frontal, occipital, parietal and temporal lobes, ($N = 168$, OLS regression, error band represents 95% CI, β for ImP and P values are reported within the respective graphs) sex is represented by pink (females) and red (males). **b,** Associations between FDG SUVR and ImP are not significant when stratified to female sex ($N = 117$, OLS regression, error band represents 95% CI, β for ImP and P values are reported within the respective graphs). **c,** FDG SUVR in males is significantly lowered in frontal and parietal lobes, but not in occipital and temporal lobes ($N = 51$, OLS regression, error band represents 95% CI, β for ImP and P values are reported within the respective graphs). **d,** FDG SUVR of males with outliers removed, shows statistically significant association only in parietal lobe ($N = 50$, OLS regression, error band represents 95% CI, β for ImP and P values are reported within the respective graphs).

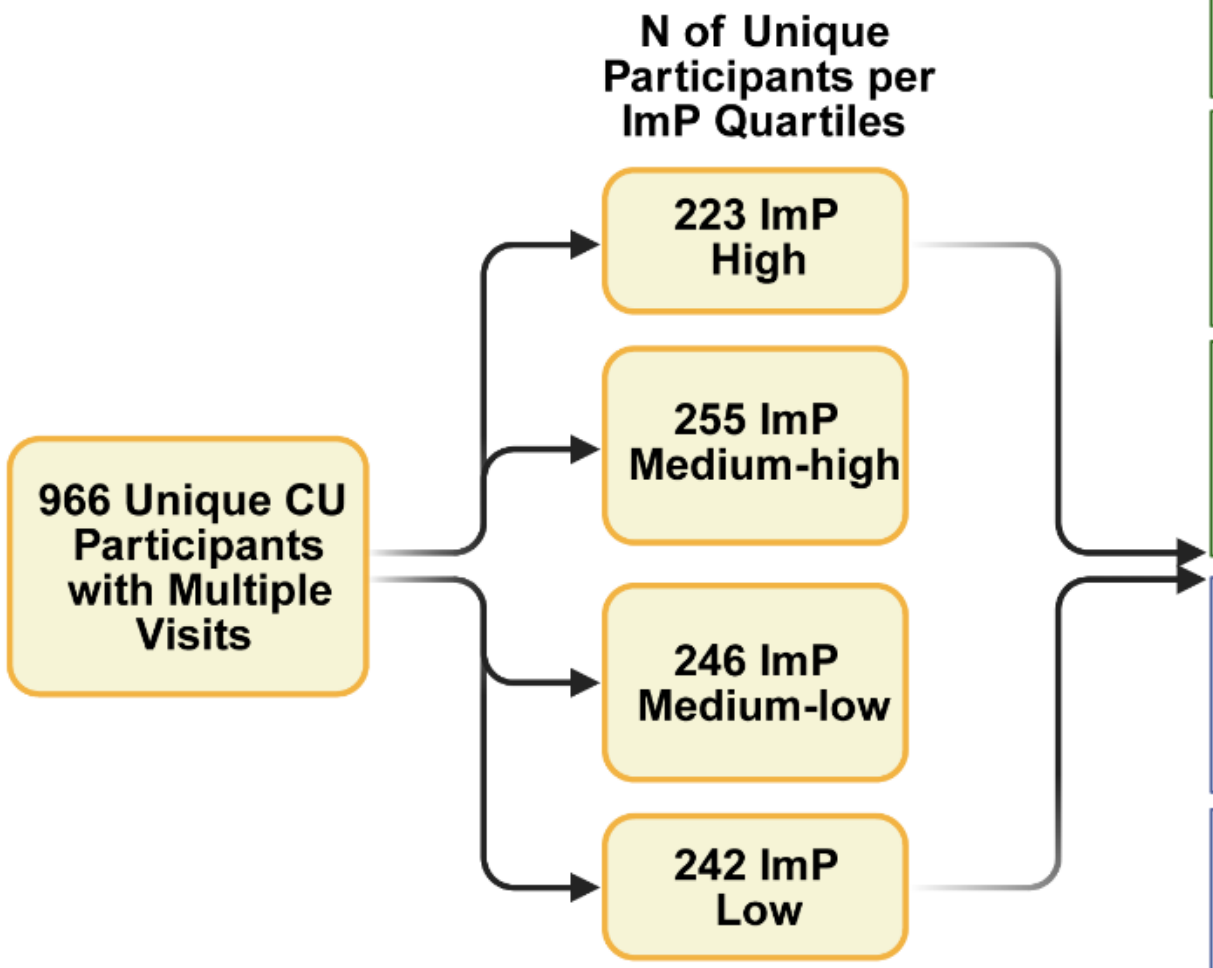

N of All Matching Rows Between Timepoints of ImP and Cognitive Scores/Plasma Biomarkers

Outcome	Outcome visit	High Visit1	High Visit2	High Visit3	Low Visit1	Low Visit2	Low Visit3
PACC3 Animal Naming	Visit1	222	221	46	242	242	61
	Visit2	218	217	46	236	236	61
	Visit3	175	184	44	213	219	58
	Visit4	87	135	39	110	169	47
	Visit5	7	49	18	14	77	26
	Visit6	NA	6	4	NA	11	5
PACC3 Letter Fluency	Visit1	223	222	46	242	242	61
	Visit2	222	221	46	242	242	61
	Visit3	214	213	46	234	234	61
	Visit4	175	175	44	211	214	58
	Visit5	102	130	38	135	167	45
	Visit6	19	55	18	23	77	26
	Visit7	NA	8	4	NA	16	5
PACC3 Trail Making Test-B	Visit1	223	222	46	242	242	61
	Visit2	221	220	46	242	242	61
	Visit3	214	212	46	235	235	61
	Visit4	171	169	43	207	210	57
	Visit5	92	108	32	130	148	43
	Visit6	12	42	14	17	61	20
	Visit7	NA	4	4	NA	7	6
pTau217	Visit1	223	222	46	241	241	61
	Visit2	222	221	46	241	241	61
	Visit3	216	214	46	235	235	61
	Visit4	175	176	44	210	213	58
	Visit5	102	131	39	136	166	45
	Visit6	19	55	18	24	79	26
	Visit7	NA	8	4	NA	16	6
NfL	Visit1	223	222	46	242	242	61
	Visit2	222	221	46	242	242	61
	Visit3	216	214	46	236	236	61
	Visit4	175	176	44	211	214	58
	Visit5	102	131	39	136	167	45
	Visit6	19	55	18	24	80	26
	Visit7	NA	8	4	NA	16	6
GFAP	Visit1	223	222	46	242	242	61
	Visit2	222	221	46	242	242	61
	Visit3	216	214	46	236	236	61
	Visit4	175	176	44	211	214	58
	Visit5	102	131	39	136	167	45
	Visit6	19	55	18	24	80	26
	Visit7	NA	8	4	NA	16	6

Supplementary Figure 4. Demogrpahic table for stratification of WRAP ADRC cohort by quartiles based on plasma ImP levels for longitudinal analysis with distributions of number of subjects represented by outcome variables.

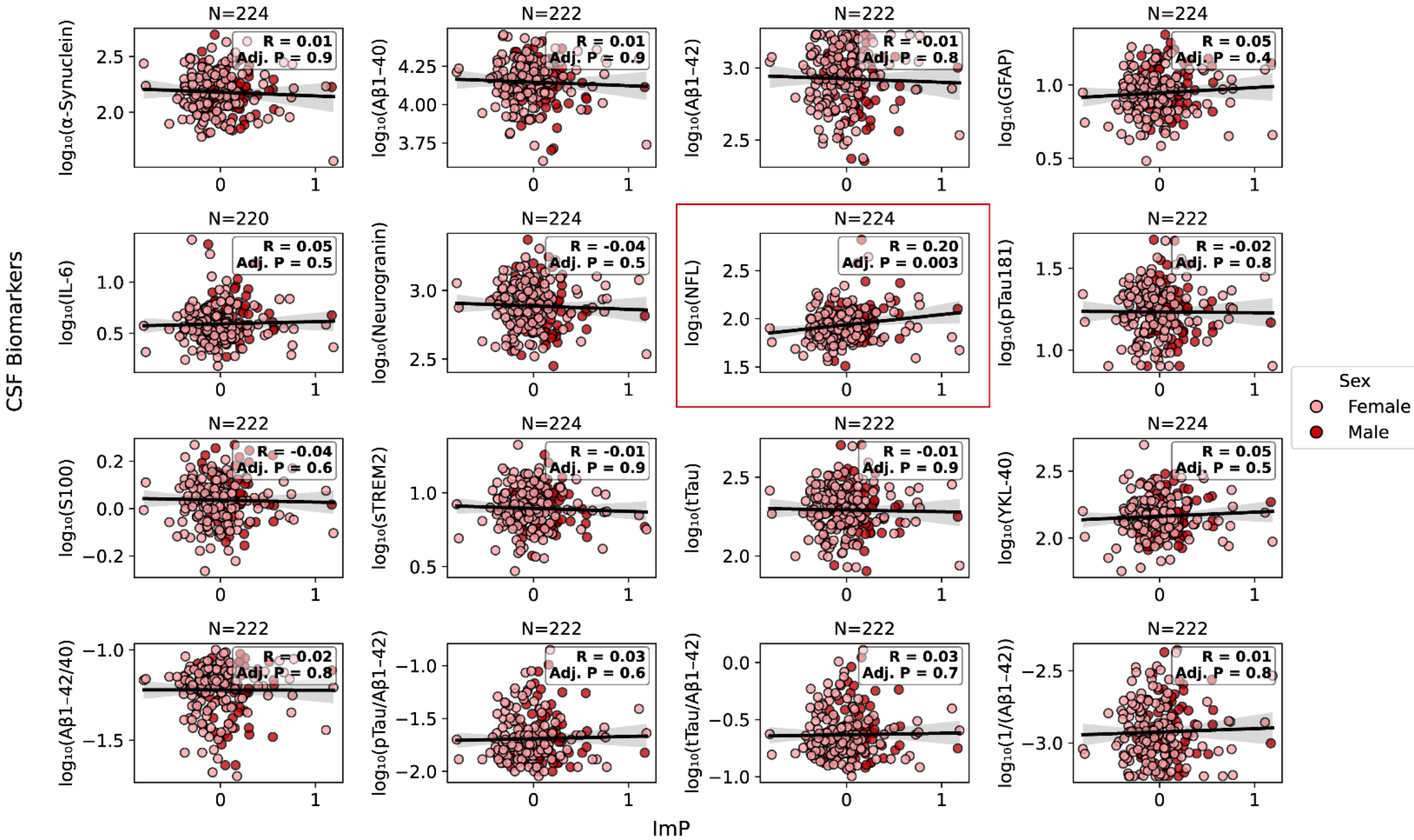

Supplementary Figure 5. Plasma ImP levels are significantly associated with CSF neurofilament light chain levels (red box) but not other CSF biomarkers of ADRD (*N*, Spearman correlation, R and FDR Adj. *P* values are reported within the respective graphs) error band represents 95% CI. Sex is represented by pink (females) and red (males)**.**

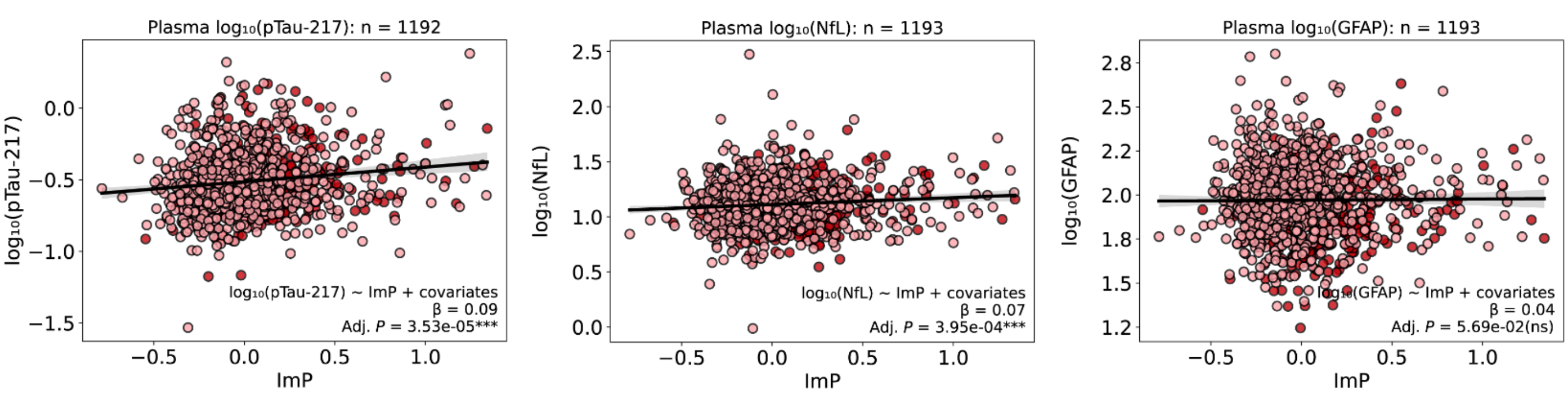

Supplementary Figure 6. Plasma ImP is associated with plasma biomarkers agnostic to comorbid LIBRA scores as a covariate. Associations were performed using OLS regression, error band represents 95% CI and Sex is represented by pink (females) and red (males) (*N*, *P*, and β [95% CI] for ImP, for respective biomarkers are reported within the graphs).

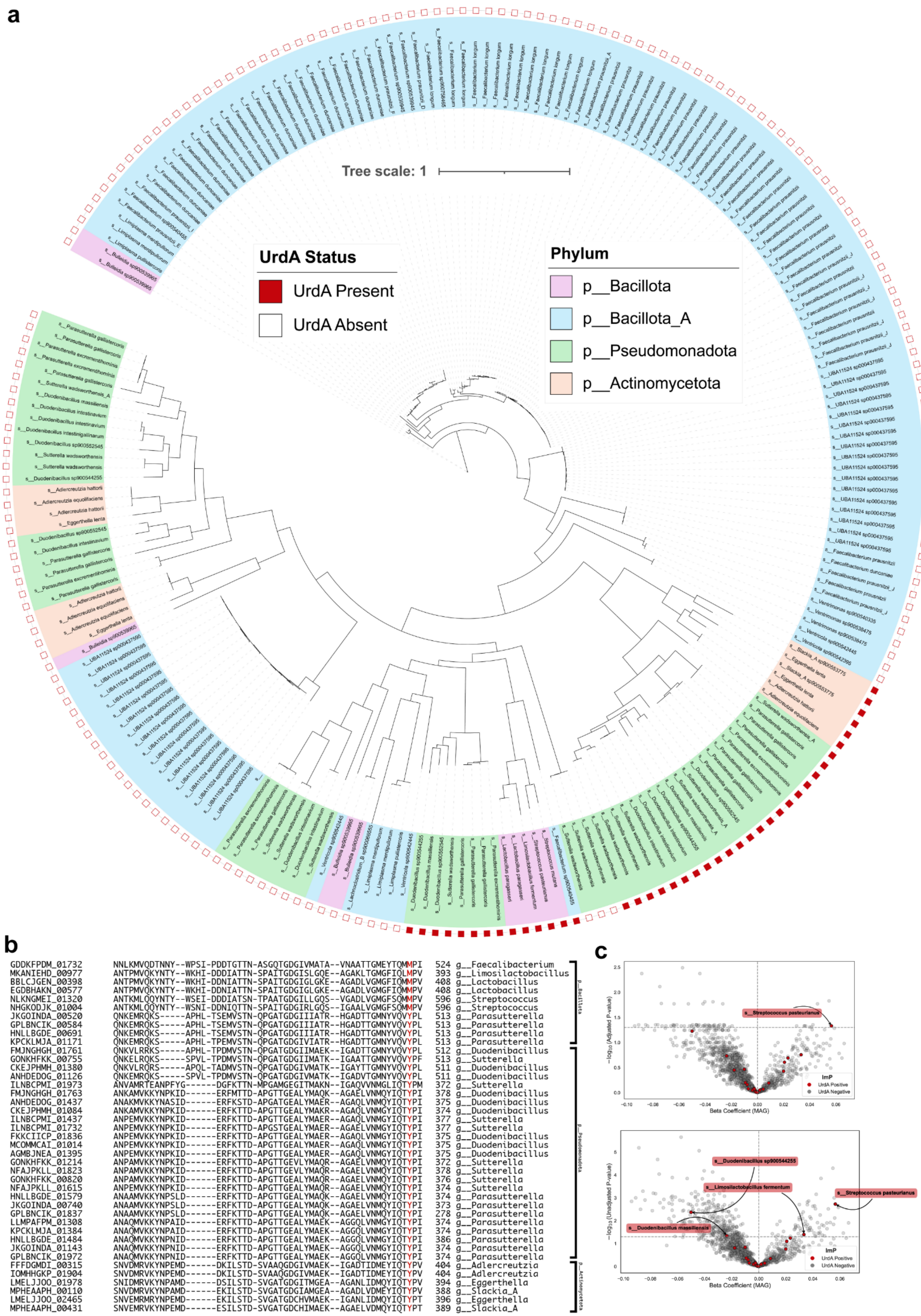
a
Tree scale: 1
UrdA Status
UrdA Present
UrdA Absent
Phylum
p__Bacillota
p__Bacillota_A
p__Pseudomonadota
p__Actinomycetota
b
GDDKFPDM_01732 NNLKMVQDTNNY--WPSI-PDDTGTTN-ASGQTGDGIVMATA--VNAATTGMEYTQMMPI 524 g__Faecalibacterium
MKANIEHD_00977 ANTPMVQKYNTY--WKHI-DDDIATTN-SPAITGDGISLGQE--AGAKLTGMGFIQLMPV 393 g__Limosilactobacillus
BBLCJGEN_00398 ANTPMVQKYNTY--WKHI-DDNIATTN-SPAITGDGIGLGKE--AGADLVGMGFIQMMPV 408 g__Lactobacillus
EGDBHAKN_00577 ANTPMVQKYNTY--WKHI-DDNIATTN-SPAITGDGIGLGKE--AGADLVGMGFIQMMPV 408 g__Lactobacillus
NLKNGMEI_01320 ANTKMLQQYNTY--WSEI-DDDIATSN-TPAATGDGILLGQS--VGADLVGMGFSQMMPV 596 g__Streptococcus
NHGKODJK_01004 ANTKMLQQYNTY--WSNI-DDNIQTTN-SPAITGDGIRLGQS--IGAALVGMGFSQMMPV 596 g__Streptococcus
JKGOINDA_00520 QNKEMRQKS-----APHL-TSEMVSTN-QPGATGDGIIIATR--HGADTTGMNYVQVYPL 513 g__Parasutterella
GPLBNCIK_00584 QNKEMRQKS-----APHL-TSEMVSTN-QPGATGDGIIIATR--HGADTTGMNYVQVYPL 513 g__Parasutterella
HNLLBGDE_00691 QNKEMRQKS-----APHL-TSEMVSTN-QPGATGDGIIIATR--HGADTTGMNYVQVYPL 513 g__Parasutterella
KPCKLMJA_01171 QNKEMRQKS-----APHL-TPEMVSTN-QPGATGDGIVIATR--HGADTTGMNYVQVYPL 513 g__Parasutterella
FMJNGHGH_01761 QNKVLRRKS-----APHL-TPDMVSTN-QPGATGDGIIMAEK--IGADTTGMNYVQVYPL 512 g__Duodenibacillus
GONKHFKK_00755 QNKELRQKS-----SPVL-TPDMVSTN-QPGATGDGIVMATK--IGAATTGMNYVQVYPF 513 g__Sutterella
CKEJPHMH_01380 QNKVLRQRS-----APQL-TADMVSTN-QPGATGDGIVMATK--IGAYTTGMNYVQVYPL 511 g__Duodenibacillus
ANHDEDOG_01126 QNKEMRQKS-----SPVL-TPDMVSTN-QPGATGDGIVMATK--HGADTTGMNYVQVYPL 511 g__Duodenibacillus
ILNBCPMI_01973 ANVAMRTEANPFYG------DGFKTTN-MPGAMGEGITMAKN--IGAQVVNMGLIQTYPM 372 g__Sutterella
FMJNGHGH_01763 ANKAMVKKYNPKID------ERFMTTD-APGATGEALYMAQK--AGAQLVNMQYIQTYPI 378 g__Duodenibacillus
ANHDEDOG_01437 ANAKMVKKYNASID------ERFKSTD-APGATGEALYMAER--AGAQLVNMQYIQTYPI 375 g__Duodenibacillus
CKEJPHMH_01084 ANKAMVKKYNPKID------ERFKTTD-APGTTGEALYMAKR--AGAQLVNMGYIQTYPI 374 g__Duodenibacillus
ILNBCPMI_01437 ANPEMVKKYNPKID------ERFKTTD-APGTTGEALYMAER--AGAELVNMQYIQTYPI 377 g__Sutterella
ILNBCPMI_01732 ANPEMVKKYNPKID------ERFKTTD-APGSTGEALYMAER--AGAELVNMQYIQTYPI 377 g__Sutterella
FKKCIICP_01836 ANPEMVKKYNPKID------ERFKTTD-APGTTGEALYMAER--AGAQLVNMQYIQTYPI 378 g__Duodenibacillus
MCOMMCAI_01014 ANPEMVKKYNPKID------ERFKTTD-APGTTGEALYMAER--AGAQLVNMQYIQTYPI 375 g__Duodenibacillus
AGMBJNEA_01395 ANPEMVKKYNPKID------ERFKTTD-APGTTGEALYMAER--AGAQLVNMQYIQTYPI 375 g__Duodenibacillus
GONKHFKK_01214 ANPAMVKKYNPKID------ERFKTTD-APGTTGEVLYMAQR--AGAELVNMQYIQTYPI 372 g__Sutterella
NFAJPKLL_01823 ANPAMVKKYNPKID------ERFKTTD-APGTTGEVLYMAQR--AGAELVNMQYIQTYPI 378 g__Sutterella
GONKHFKK_00820 ANPAMVKKYNPKID------ERFKTTD-APGTTGEALYMAQR--AGAELVNMQYIQTYPI 376 g__Sutterella
NFAJPKLL_01615 ANPEMVKKYNPKID------ERFKTTD-APGTTGEALYMAER--AGAELVNMQYIQTYPI 374 g__Sutterella
HNLLBGDE_01579 ANAAMVKKYNPSLD------ERFKTTD-APGTTGEALYMAQK--AGAELVNMQYIQTYPI 374 g__Parasutterella
JKGOINDA_00740 ANAAMVKKYNPSLD------ERFKTTD-APGTTGEALYMAQK--AGAELVNMQYIQTYPI 373 g__Parasutterella
GPLBNCIK_01837 ANAAMVKKYNPSLD------ERFKTTD-APGTTGEALYMAQK--AGAELVNMQYIQTYPI 278 g__Parasutterella
LLMPAFPM_01308 ANAQMVKKYNPAID------ERFKTTD-APGTTGEALYMAEK--AGGQLVNMGYIQTYPI 374 g__Parasutterella
KPCKLMJA_01384 ANAQMVKKYNPAID------ERFKTTD-APGTTGEALYMAEK--AGGQLVNMGYIQTYPI 374 g__Parasutterella
HNLLBGDE_01484 ANAQMVKKYNPNID------ERFKTTD-APGTTGEALYMAEK--AGGQLVNMGYIQTYPI 386 g__Parasutterella
JKGOINDA_01143 ANAQMVKKYNPNID------ERFKTTD-APGTTGEALYMAEK--AGGQLVNMGYIQTYPI 374 g__Parasutterella
GPLBNCIK_01972 ANAQMVKKYNPNID------ERFKTTD-APGTTGEALYMAEK--AGGQLVNMGYIQTYPI 374 g__Parasutterella
FFFDGMDI_00315 SNVDMRVKYNPEMD------DKILSTD-SVAAQGDGIVMAEK--IGADTIDMEYIQTYPV 404 g__Adlercreutzia
IOMHHGKP_01904 SNVDMRVKYNPEMD------DKILSTD-SVAAQGDGIVMAEK--IGADTIDMEYIQTYPV 404 g__Adlercreutzia
LMELJJOO_01978 SNIDMRVKYNPAMD------DSILSTD-SVGATGDGITMGEA--IGANLIDMQYIQTYPV 394 g__Eggerthella
MPHEAAPH_00110 SNVDMRVKYNPAMD------EKILSTD-SVGATGDGIAMGEA--AGANLVDMEYIQTYPV 388 g__Slackia_A
LMELJJOO_02465 SNVEMRMRYNPEMD------EKILSTD-SVGATGDGITMGEA--IGANLIDMQYIQTYPT 396 g__Eggerthella
MPHEAAPH_00431 SNVEMRVKYNPEMD------ESILSTD-SVGATGDCHIMAEK--AGAELVDMQYIQTYPT 389 g__Slackia_A
p__Bacillota
p__Pseudomonadota
p__Actinomycetota
c
s__Streptococcus pasteurianus
−log10(Adjusted P-value)
Beta Coefficient (MAG)
ImP
UrdA Positive
UrdA Negative
s__Duodenibacillus sp900544255
s__Limosilactobacillus fermentum
s__Streptococcus pasteurianus
s__Duodenibacillus massiliensis
−log10(Unadjusted P-value)
Beta Coefficient (MAG)
ImP
UrdA Positive
UrdA Negative

Supplementary Figure 7. Fecal metagenomics revealed several gut bacterial taxa harboring putative *urdA* orthologs. **a,** Full sized phylogenetic tree depicting the various MAGs within MARS cohort (N = 294). Each terminal branch represents a species, colored by phylum. The outer layer of the tree indicates the presence (red) or absence (white) of the *urdA* gene. **b,** Representative *urdA* sequences marked with 373rd amino acid in red. **c,** mPACC3 scores AN and CFL are negatively associated with *urdA*$^{+}$ taxa (Top). *Streptococcus pasteurianus* carrying putative *urdA* ortholog is positively associated with plasma ImP levels (Bottom) (*P*-value, FDR Adj. *P*-value, and β-coefficient were determined using OLS regression).

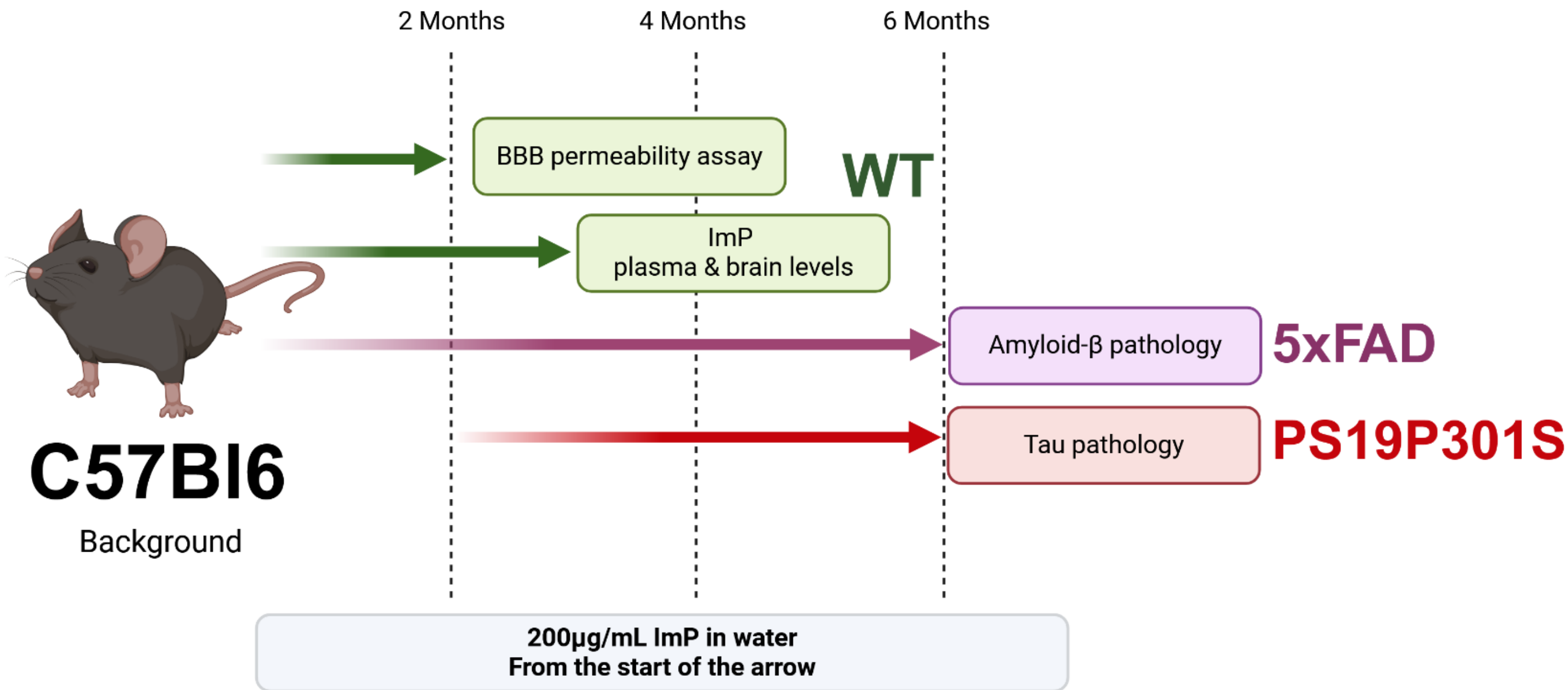

Supplementary Figure 8. Mouse models employed to tests causal effects of ImP on ADRD-like pathology. Mice were treated with ImP supplemented in drinking water WT and 5xFAD were started at weaning and PS19P301S mice were started in ImP at 2 months of age. Created in BioRender. Kuehn, J. (2026) https://BioRender.com/3oifs9f.

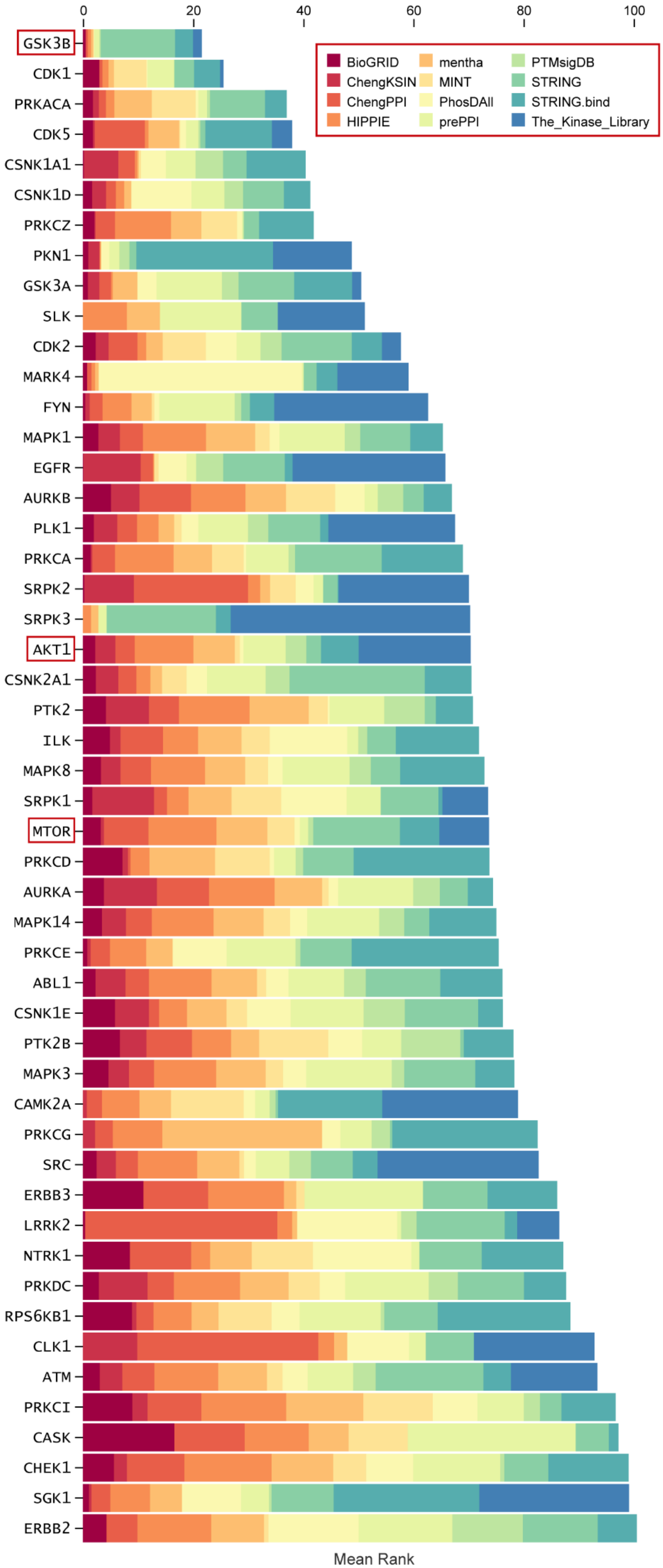

0
20
40
60
80
100
GSK3B
CDK1
PRKACA
CDK5
CSNK1A1
CSNK1D
PRKCZ
PKN1
GSK3A
SLK
CDK2
MARK4
FYN
MAPK1
EGFR
AURKB
PLK1
PRKCA
SRPK2
SRPK3
AKT1
CSNK2A1
PTK2
ILK
MAPK8
SRPK1
MTOR
PRKCD
AURKA
MAPK14
PRKCE
ABL1
CSNK1E
PTK2B
MAPK3
CAMK2A
PRKCG
SRC
ERBB3
LRRK2
NTRK1
PRKDC
RPS6KB1
CLK1
ATM
PRKCI
CASK
CHEK1
SGK1
ERBB2
BioGRID
ChengKSIN
ChengPPI
HIPPIE
mentha
MINT
PhosDAll
prePPI
PTMsigDB
STRING
STRING.bind
The_Kinase_Library
Mean Rank

Supplementary Figure 9. Kinase enrichment analysis of the top 25 differentially abundant phosphoproteins from PS19P301S cortical lysates, shown as stacked bar plots for top 100 highest-ranked kinases. Specific kinases of interest are marked with a red box.

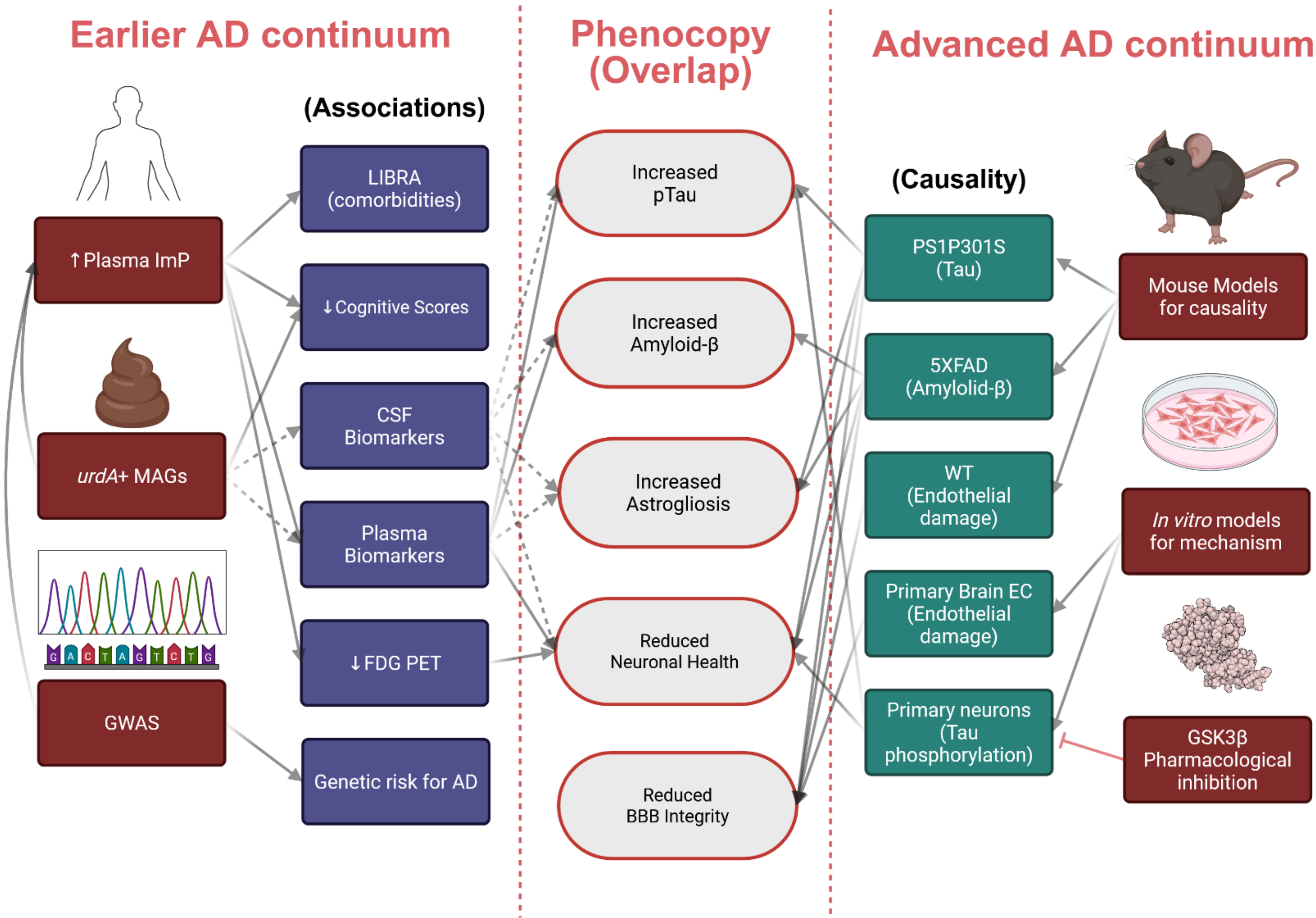

Supplementary Figure 10. Simplified study schematic illustrating observations (dotted arrows indicate supporting trends) from clinical datasets that include individuals in earlier stages of AD continuum (cognitively unimpaired), and GWAS study which associates ImP with AD. We also found that ImP is causal to exacerbated hallmark AD advanced-stage neuropathological features in preclinical models. Overall, we identify multiple lines of evidence linking the gut-bacterial metabolite ImP to AD. Continuous lines show statistically significant evidence whereas dotted lines show supporting trends. Created in BioRender. Kuehn, J. (2026) https://BioRender.com/lk7w0kg.

Supplementary Methods

Plasma sample collection and ImP analysis

Fasting blood samples were drawn from study participants from the WRAP study as previously described[1,2]. Plasma samples were obtained from the blood samples by centrifugation at 3000 revolutions per minute for 15 minutes at room temperature. Plasma levels of ImP were determined by HPLC/MS (Metabolon, Inc.), as previously described[3]. The raw metabolite data was median scaled, log-transformed, and K-Nearest Neighbors (KNN) imputation was applied to fill in any missing metabolite values using the KNNImputer from the sklearn.impute module (v1.4.1), with five nearest neighbors. The imputed ImP data was used for downstream analyses.

Plasma biomarkers pTau-217 and NfL

The commercial ALZpath pTau-217 assay was used on the Simoa® platform to analyze plasma pTau-217 levels from WRAP participants. Plasma biomarkers, including NfL, from WRAP were analyzed and quantified using the commercial Neurology 4-plex E kit (103670; Quanterix). All assays were performed at the Department of Psychiatry and Neurochemistry, University of Gothenburg[4].

Analysis of cognitive functions with mPACC3 scores

Three composite tests for measuring executive functions, along with assessments of two other cognitive domains—immediate learning and delayed recall—were used to compute the global composite scores for the three-test version of the Preclinical Alzheimer's Cognitive Composite (PACC3). The PACC3 scores were derived using three distinct measures of executive functions—Animal Naming Test (PACC3-AN), Category Fluency Test (PACC3-CFL), and Trail-Making Test B (PACC3-TRLB)—and subsequently transformed into z-scores[5]. The modified Preclinical Alzheimer's Cognitive Composite (mPACC3) scores were used for all associations pertaining to cognitive function. The mPACC was adapted to address sampling limitations during the COVID-19 pandemic with remote assessments[6]. The test battery included telemetric animal naming (mPACC3-AN), categoric fluency or letter fluency (mPACC3-CFL) tests. Additionally, we used in-person trail making test-B (mPACC3-TRL-B) results to include all available overlapping data without selection bias.

CSF biomarkers

Cerebrospinal fluid (CSF) samples were collected via lumbar puncture in the morning after an 0-12 hour fast, as previously described[7]. CSF biomarkers were measured using the NeuroToolKit (NTK), a panel of exploratory robust prototype assays (Roche Diagnostics International Ltd, Rotkreuz, Switzerland). The following biomarkers were measured as markers of AD and related pathologies and quantified on the Cobas® e 601 module (Roche Diagnostics International Ltd, Rotkreuz, Switzerland): $A\beta_{42}$, $pTau_{181}$, tTau, S100 calcium-binding protein B (S100B), and interleukin-6 (IL-6). The remaining biomarkers were assayed on the Cobas® e 411 analyzer: $A\beta_{40}$, neurofilament light protein (NFL), neurogranin, α-synuclein, glial fibrillary acidic protein (GFAP), chitinase-3-like protein 1 (YKL-40), and soluble triggering receptor expressed on myeloid cells 2 (sTREM2).

FDG PET imaging

FDG PET imaging was performed using a Siemens EXACT HR+ tomograph in 3D mode at the Waisman Center Brain Imaging Laboratory, University of Wisconsin-Madison[8]. Prior to scanning, participants were screened to confirm glucose levels below 180 mg/dL. Imaging began with a 6-minute transmission scan for attenuation correction, followed by a 30-minute dynamic emission scan (6 × 5-minute frames) starting 30 minutes after administering a bolus injection of approximately 185 MBq of [18F]FDG. PET data were reconstructed using filtered back-projection, with corrections for random events, scatter, segmented attenuation, dead time, scanner normalization, and radioactive decay. The reconstructed images had a matrix size of 128 × 128 × 63 with voxel dimensions of 2.57 × 2.57 × 2.43 mm. PET images were processed using a custom MATLAB script. The reconstructed PET time series were smoothed with an isotropic 6 mm Gaussian kernel, realigned between frames using SPM12, and summed for the 30–60-minute post-injection period. Parametric standard uptake value ratio (SUVR) images were created by linearly aligning the summed PET image to the corresponding T1-weighted magnetic resonance imaging (MRI) and normalizing the intensities with cerebellar gray matter as the reference region. Regional mean FDG SUVR values were extracted from bilateral regions of interest (ROIs), including the frontal cortex, lateral temporal cortex (encompassing the middle and superior temporal gyri), supramarginal and angular gyri, precuneus, posterior cingulate, and hippocampus. The selection of these regions for analysis was based on earlier studies that identified reduced FDG uptake in individuals with AD dementia[9,10].

Shotgun sequencing

DNA library samples were prepared using Illumina NexteraXT library preparation kit. Quality and quantity of the finished libraries were assessed using an Agilent bioanalyzer and Qubit® dsDNA HS Assay Kit, respectively. Libraries were standardized to 2nM. Paired-end, 150 bp sequencing was performed using the Illumina NovaSeq6000, producing 2x150bp

paired-end reads. Images were analyzed using the standard Illumina Pipeline (v1.8.2). Low-quality reads were filtered out from raw DNA reads using Trimmomatic (v0.39) with parameters (SLIDINGWINDOW:4:20 MINLEN:50). To identify and eliminate host sequences, reads were aligned against the Homo sapiens genome (GRCh38, Rel109) using bowtie2 (v2.3.4) with default settings, and microbial DNA reads that did not align with the human genome were identified using samtools (v1.3) (samtools view -b -f 4 -f 8). Shotgun metagenomic data are available from the Sequence Read Archive (SRA) under accession number PRJNA1271016.

Proteomics and phosphoproteomics

Flash frozen brain cortices from 4 PS19 P301S male mice were homogenized in extraction buffer (25mM Tris HCl, 150mM NaCl, 10mM EDTA, 10mM EGTA, 1mM DTT, protease and phosphatase inhibitors) using ceramic beads and a bead-beater. Lysates were centrifuged at 16,000 x g for 20 min at 4°C and supernatants were collected. 200µL lysates were transferred to 2mL low-protein binding microcentrifuge tubes and 800µL of LC-MS grade methanol was added (80% final v/v) for BSL2 inactivation in biosafety cabinet by vortexing and incubation at room temperature for 5min. Subsequently 250µL chloroform was added, followed by heavy vortexing and 600µL water with heavy vortexing to facilitate protein precipitation and two-phase partitioning. Samples were spun down for 1min at max speed at room temperature and upper-phase was removed carefully not to disturb the protein interphase. 1mL methanol was added, samples were vortexed and spun for 4 minutes to pellet the protein, supernatant was removed and another round of protein extraction on second 200µL aliquot of lysate was done in the same tube to a total volume of 400µL lysate. Two additional cold acetone washes were performed by repeating aforementioned vortex and centrifuge steps in between and finally pellets were air dried to completion. Dried pellets were re-solubilized in 65µL of 8M Urea in 50mM NH4HCO3 (pH8.5) and 5µL was taken for BCA protein measurement. Subsequently 100µg of protein in 30µL 8M urea and 50mM NH4HCO3 (pH8.5) per each sample was taken for tryptic/LysC digestion where the samples were first diluted with 5µl of 25mM DTT and 25mM NH4HCO3 (pH8.5) to 120µL final volume for the reduction step, which was carried out for 15min at 56°C. After cooling on ice to room temperature 6µL of 55mM CAA (chloroacetamide) was added for alkylation where samples were incubated in darkness at room temperature for 15 minutes. This reaction was quenched by 16µL addition of 25mM DTT. Subsequently 40uL of trypsin/LysC solution consisting of 100ng/µL 1:1 Trypsin [Promega]:LysC [FujiFilm] mix in 25mM NH4HCO3 along with 18µL of 25mM NH4HCO3 (pH8.5) was added to the samples for a final 200µL volume. Digests were carried out overnight at 37°C then subsequently terminated by acidification with 30µL of 2.5% TFA (Trifluoroacetic Acid) to 0.32% final plus 3µL of 10% HFBA (Heptafluorobutyric acid) to 0.13% final and samples were desalted and concentrated using 10mg/1ml Strata™-X 33µm Polymeric Reverse Phase SPE cartridges [Phenomenex] per manufacturer protocol. Eluates in 70%:28%:2% acetonitrile:water:formic acid were dried to completion then reconstituted in 100µL of 100mM TEAB (Triethylammonium Bicarbonate) for TMT labelling with 40µL [20µg/µL] of reagent [TMT 10plex™ Labeling Reagent Set Lot#AA401548A from Thermo Scientific in 100% acetonitrile] done at room temperature for 1hr with intermittent gentle vortexing every 15min. The labeling reaction was terminated with 8µl addition of 5% hydroxylamine [0.27% final] and 15min incubation at room temperature. ~1/37th (4µL, ~2.6µg) of each individually labelled sample was taken and pooled together for a test pool run to evaluate complexity and mixing ratio. Post test-pool evaluation, master-pool sample was generated where each biological sample was present at the same total protein mass (~1mg total protein in ~1.1mL total volume), acidified to 1% formic acid (pH~3), frozen (-80ºC) then dried to almost completion in the speed vac. Subsequently resolubilized in 600µL of 5% acetonitrile, 0.3% TFA and 0.2% HFBA for solid-phase extraction using 30mg Strata™-X 33µm Polymeric Reverse Phase SPE cartridge [Phenomenex] per manufacturer protocol. 1/24th of eluate was used for the total protein profiling and the rest was retained for the phosphoenrichment where the eluate in 75/23/2% Acetonitrile/H2O/Formic acid was dried to completion in the speed-vac then brought back to 200µL total volume with 1M glycolic acid in 80% Acetonitrile and 5% Trifluoroacetic acid immediately before phosphoenrichment, which was carried out in two stages, first using 5mg of titanium dioxide magnetic microparticles [MagReSyn® TiO2 from ReSyn Biosciences] then unbound material was enriched with 2mg of Zirconium-ion (Zr4+) functional magnetic microparticles [MagReSyn® Zr-IMAC from ReSyn Biosciences] according to manufacturer protocol. Eluates from sequential enrichments were combined and dried in the speed-vac to completion. Sample handling for both enrichments were according to the manufacturer protocols. Enriched samples were solubilized in 35µL of 0.3% TFA, 0.2% HFBA and 3% ACN then desalted using 10µL Pierce® C18 Tips [Thermo Fisher Scientific] according to manufacturer protocol. Eluates in 75%:25%:0.1%

acetonitrile:water:TFA acid (v/v) were dried to completion in the speed-vac and reconstituted in 15µL of 0.1% formic acid and 5% acetonitrile for injection on the instrument.

NanoLC-MS/MS

Peptides were analyzed on Orbitrap Fusion™ Lumos™ Tribrid™ platform, where 3µL [~2.4µg] total proteome or 3µL [1/5th] total enriched phospho-proteome was injected using Dionex UltiMate™3000 RSLCnano delivery system [ThermoFisher Scientific] equipped with an EASY-Spray™ electrospray source (held at constant 50°C). Chromatography of peptides prior to mass spectral analysis was accomplished using capillary emitter column [PepMap® C18, 2µM, 100Å, 500 x 0.075mm, Thermo Fisher Scientific]. NanoHPLC system delivered solvents A: 0.1% (v/v) formic acid , and B: 80% (v/v) acetonitrile, 0.1% (v/v) formic acid at 0.30 µL/min to load the peptides at 2% (v/v) B, followed by quick 2 minute gradient to 5% (v/v) B and gradual analytical gradient from 5% (v/v) B to 62.5% (v/v) B over 203 min when it concluded with rapid 10 minute ramp to 95% (v/v) B for a 9 minute flash-out. As peptides eluted from the HPLC-column/electrospray source survey MS scans were acquired in the Orbitrap with a resolution of 60,000, max inject time of 50ms and AGC target of 1,000,000 followed by HCD-type MS2 fragmentation into Orbitrap (36% collision energy and 30,000 resolution) with 0.7 m/z isolation window in the quadrupole under ddMSnScan 1 second cycle time mode with peptides detected in the MS1 scan from 400 to 1400 m/z with max inject time of 54ms and AGC target of 125,000; redundancy was limited by dynamic exclusion and MIPS filter mode ON.

Protein enrichment

Protein enrichment was performed using differentially regulated hits (>±30% FC) that are statistically significant (FDR $P<0.05$) on Metascape[11] to obtain direct overlaps, functional overlaps, and pathway overlaps between 5XFAD and PS19P301S models. To sort phosphoproteome by interacting kianses, we enriched top-25 (>30% FC) phosphoproteins using Kinase Enrichment Analysis 3 (KEA3)[12] and cell compartment enrichment was performed using Enrichr[13–16].

REFERENCES

1. Johnson, S. C. *et al.* The Wisconsin Registry for Alzheimer's Prevention: A review of findings and current directions. *Alzheimers Dement (Amst)* **10**, 130–142 (2017).
2. Bridgewater Br, E. A. High Resolution Mass Spectrometry Improves Data Quantity and Quality as Compared to Unit Mass Resolution Mass Spectrometry in High-Throughput Profiling Metabolomics. *Metabolomics* **04**, (2014).
3. Darst, B. F., Koscik, R. L., Hogan, K. J., Johnson, S. C. & Engelman, C. D. Longitudinal plasma metabolomics of aging and sex. *Aging (Albany NY)* **11**, 1262–1282 (2019).
4. Ashton, N. J. *et al.* Diagnostic Accuracy of a Plasma Phosphorylated Tau 217 Immunoassay for Alzheimer Disease Pathology. *JAMA Neurology* **81**, 255–263 (2024).
5. Jonaitis, E. M. *et al.* Measuring longitudinal cognition: Individual tests versus composites. *Alzheimer's & Dementia: Diagnosis, Assessment & Disease Monitoring* **11**, 74–84 (2019).
6. Jonaitis, E. M. *et al.* Validating remote cognitive tests in the Wisconsin Registry for Alzheimer's Prevention. *Alzheimer's & Dementia: Diagnosis, Assessment & Disease Monitoring* **17**, e70153 (2025).

7. Van Hulle, C. *et al.* An examination of a novel multipanel of CSF biomarkers in the Alzheimer's disease clinical and pathological continuum. *Alzheimer's & Dementia* **17**, 431–445 (2021).
8. Ennis, G. E. *et al.* The relationship of glucose-stimulated insulin secretion to cerebral glucose metabolism and cognition in healthy middle-aged and older adults. *Neurobiology of Aging* **105**, 174–185 (2021).
9. Langbaum, J. B. S. *et al.* Categorical and correlational analyses of baseline fluorodeoxyglucose positron emission tomography images from the Alzheimer's Disease Neuroimaging Initiative (ADNI). *NeuroImage* **45**, 1107–1116 (2009).
10. Mosconi, L. *et al.* FDG-PET changes in brain glucose metabolism from normal cognition to pathologically verified Alzheimer's disease. *Eur J Nucl Med Mol Imaging* **36**, 811–822 (2009).
11. Zhou, Y. *et al.* Metascape provides a biologist-oriented resource for the analysis of systems-level datasets. *Nat Commun* **10**, 1523 (2019).
12. Kuleshov, M. V. *et al.* KEA3: improved kinase enrichment analysis via data integration. *Nucleic Acids Res* **49**, W304–W316 (2021).
13. Binder, J. X. *et al.* COMPARTMENTS: unification and visualization of protein subcellular localization evidence. *Database (Oxford)* **2014**, bau012 (2014).
14. Chen, E. Y. *et al.* Enrichr: interactive and collaborative HTML5 gene list enrichment analysis tool. *BMC Bioinformatics* **14**, 128 (2013).
15. Xie, Z. *et al.* Gene Set Knowledge Discovery with Enrichr. *Current Protocols* **1**, e90 (2021).
16. Kuleshov, M. V. *et al.* Enrichr: a comprehensive gene set enrichment analysis web server 2016 update. *Nucleic Acids Res* **44**, W90-97 (2016).
