# Supplementary material for: Gut bacterial metabolite imidazole propionate potentiates Alzheimer’s disease pathology
Source: Nat Commun. 2026 Jun 26;17:8017. doi: 10.1038/s41467-026-74744-z (PMC13454616; doi:10.1038/s41467-026-74744-z)
Supplement: Supplementary file 3 [file 41467_2026_74744_MOESM9_ESM.pdf]

Corresponding author(s): Federico Rey

Last updated by author(s): 2026/05/27

Reporting Summary

Nature Portfolio wishes to improve the reproducibility of the work that we publish. This form provides structure for consistency and transparency in reporting. For further information on Nature Portfolio policies, see our Editorial Policies and the Editorial Policy Checklist.

Statistics

For all statistical analyses, confirm that the following items are present in the figure legend, table legend, main text, or Methods section.

n/a	Confirmed	
☐	☒	The exact sample size (*n*) for each experimental group/condition, given as a discrete number and unit of measurement
☐	☒	A statement on whether measurements were taken from distinct samples or whether the same sample was measured repeatedly
☐	☒	The statistical test(s) used AND whether they are one- or two-sided<br>*Only common tests should be described solely by name; describe more complex techniques in the Methods section.*
☐	☒	A description of all covariates tested
☐	☒	A description of any assumptions or corrections, such as tests of normality and adjustment for multiple comparisons
☐	☒	A full description of the statistical parameters including central tendency (e.g. means) or other basic estimates (e.g. regression coefficient) AND variation (e.g. standard deviation) or associated estimates of uncertainty (e.g. confidence intervals)
☐	☒	For null hypothesis testing, the test statistic (e.g. *F*, *t*, *r*) with confidence intervals, effect sizes, degrees of freedom and *P* value noted<br>*Give P values as exact values whenever suitable.*
☒	☐	For Bayesian analysis, information on the choice of priors and Markov chain Monte Carlo settings
☒	☐	For hierarchical and complex designs, identification of the appropriate level for tests and full reporting of outcomes
☐	☒	Estimates of effect sizes (e.g. Cohen's *d*, Pearson's *r*), indicating how they were calculated

Our web collection on statistics for biologists contains articles on many of the points above.

Software and code

Policy information about availability of computer code

Data collection	3 images were taken from random regions within retrosplenial cortex in 5XFAD mice and piriform cortex in PS19P301S mice, from 3 different coronal sections, and 20-24 images were taken from neurons within primary neuronal cultures, enriched for isolated neurons with images centered to the nucleus, using Zeiss LSM 800 (UW-Madison, Medical Microbiology and Immunology) and Nikon AlR microscopes (UW-Madison Biochemistry Optical Imaging Core); lmaris v9.5.l (Oxford Instruments) with MATLAB (Mathworks) XTensions; SNT Neuroanatomy plugin on image J; nanoLC-MS/MSon an Orbitrap Fusion Lumas platform for proteomics and phosphoproteomics; Additional details in "Methods" section and "Supplementary materials and methods".
Data analysis	SPAdes (v.3.15.5; metaspades.py-k 21,33,55,77), Bowtie2 401 (v.2.3.4), MetaBAT2 (v.2.17), CheckM (v.1.2.2), dRep (v.3.5.0; -pa 0.9-405 sa 0.99), Genome Taxonomy Database Toolkit (GTDB-Tk; v.2.3.2), GTDB database (ver. R214), Prodigal (v.2.6.3), kofamscan (v.1.3.0), Carbohydrate-Active Enzymes(CAZymes) database, dbcan3 409 (v.4.1.4), MUSCLE (v.3.8.31), HMMER (v.3.2.1), HMM search (v.3.2.1), Clustal Omega, ShinyGO v0.82, Metascape v3.5.20250701, Python (v3.12.1), SciPy 496 (vl.11.4), statsmodels (v0.14.1), pandas v2.2.1, Matplotlib (v3.8.0), seaborn (v0.13.2), Biopython (vl.85), Interactive Tree of Life (iTOL, v7.1), GraphPad [PRISM 537 v.10.3.1(509)], Proteome Discoverer 543 3.1.0.638 and Seaquest HT search 544 engine platform. Additional details in "Methods" section and "supplementary materials and methods".

For manuscripts utilizing custom algorithms or software that are central to the research but not yet described in published literature, software must be made available to editors and reviewers. We strongly encourage code deposition in a community repository (e.g. GitHub). See the Nature Portfolio guidelines for submitting code & software for further information.

Data

Policy information about availability of data

All manuscripts must include a data availability statement. This statement should provide the following information, where applicable:

- Accession codes, unique identifiers, or web links for publicly available datasets
- A description of any restrictions on data availability
- For clinical datasets or third party data, please ensure that the statement adheres to our policy

All clinical data are available in previous publications mentioned in the respective methods section. The metagenomics raw sequences included in this study were deposited in the Sequence Read Archive (SRA) under accession number PRJNA1271016. The metagenomic assemblies (MAGs) generated for this study were deposited in the SRA under accession number PRJNA1469852. Proteomics and phosphoproteomics data is available via ProteomeXchange with identifier PXD077817.

Research involving human participants, their data, or biological material

Policy information about studies with human participants or human data. See also policy information about sex, gender (identity/presentation), and sexual orientation and race, ethnicity and racism.

Reporting on sex and gender	Sex (male/female) was recorded based on self-report at enrollment. Analyses included sex as a covariate or stratification variable, and sex-specific results are reported where relevant. Gender identity was not separately assessed.
Reporting on race, ethnicity, or other socially relevant groupings	Race was self-reported at baseline. The study sample was predominantly White (94%), with limited representation from other racial or ethnic groups. Due to small sample sizes in non-White subgroups, race was not included as a covariate in statistical models. Race distributions are reported in the demographic table. Ethnicity (e.g., Hispanic/Latino) and other social groupings were not separately assessed in this dataset.
Population characteristics	Participants were enrolled through the Wisconsin Alzheimer's Disease Research Center (ADRC), the Wisconsin Registry for Alzheimer's Prevention (WRAP), and the Microbiome in Alzheimer's Risk Study (MARS). The cohort included in the analysis was cognitively unimpaired individuals (ImP), and some participants met criteria for mild cognitive impairment (MCI) or Alzheimer's disease (AD), who were included in the metagenomics analysis. Age, sex, race, diagnosis, APOE ε 4 carrier status, and body mass index (BMI) were recorded. Key demographic variables are summarized in the manuscript's Extended Data tables 1 and 2.
Recruitment	Participants were recruited through ongoing observational cohort studies conducted at the University of Wisconsin-Madison. WRAP primarily enrolled cognitively unimpaired, middle-aged adults enriched for a parental history of Alzheimer's disease. The ADRC cohort included a broader age range and diagnostic spectrum. The MARS study was designed to examine the gut microbiome in relation to Alzheimer's disease risk and enrolled participants from WRAP and ADRC who consented to provide stool samples and related data. Written informed consent was obtained from all participants under Institutional Review Board (IRB)-approved protocols.
Ethics oversight	All procedures involving human participants were approved by the University of Wisconsin-Madison Health Sciences IRB. Participants provided written informed consent prior to participation in the parent cohorts (WRAP or ADRC) and for the MARS study.

Note that full information on the approval of the study protocol must also be provided in the manuscript.

Field-specific reporting

Please select the one below that is the best fit for your research. If you are not sure, read the appropriate sections before making your selection.

☒ Life sciences ☐ Behavioural & social sciences ☐ Ecological, evolutionary & environmental sciences

For a reference copy of the document with all sections, see nature.com/documents/nr-reporting-summary-flat.pdf

Life sciences study design

All studies must disclose on these points even when the disclosure is negative.

Sample size	Sample sizes were determined from previous published literature.
Data exclusions	1 data point from Figure. 6e was excluded due to abnormally high dye pooling in brain of a test mouse. Including/testing this would drastically support our hypothesis. However, it would not be reproducible.
Replication	All mice experiments were performed with littermates split equally into treatment and control groups. Experiments were first performed at and n=4 and latter repeated with an additional n=4. For PS19P301S mice, the second batch of n=4 were obtained from a different colony from the

	vendor. This addresses cage, colony and lineage effects up to an extent.
Randomization	littermates split equally into treatment and control groups immediately after weaning
Blinding	images were blinded using an in-house bash script prior to analysis.

Reporting for specific materials, systems and methods

We require information from authors about some types of materials, experimental systems and methods used in many studies. Here, indicate whether each material, system or method listed is relevant to your study. If you are not sure if a list item applies to your research, read the appropriate section before selecting a response.

Materials & experimental systems

n/a	Involved in the study
☐	☒ Antibodies
☐	☒ Eukaryotic cell lines
☒	☐ Palaeontology and archaeology
☐	☒ Animals and other organisms
☐	☒ Clinical data
☒	☐ Dual use research of concern
☒	☐ Plants

Methods

n/a	Involved in the study
☒	☐ ChIP-seq
☒	☐ Flow cytometry
☒	☐ MRI-based neuroimaging

Antibodies

Antibodies used	(Anti-GFAPCatalog: 53-9892-82; 488 conjugated; eBioscience; Lot :2892568) (Anti-AT-8 Catalog: MN1020; Lot: AA46025650) (Anti-MAPP2; Abeam Catalog# ab5392) (Phospho-Tau (Thr217) Polyclonal Antibody; lnvitrogen Catalog# 44-744)
Validation	GFAP: QC: https://assets.thermofi sher.com/TFS-Assets%2FLSG%2 Fcertificate%2FCertificate-of-Analysis%2F53-9892-82 _2892568.PDF AT-8: QC: https://assets.thermofi sher.com/TFS-Assets%2Fcertificate%2FMBN%2FCOA% 2FCOA_MN1020_AA46025650_1.pdf Antibodies in cell culture experiments (primary neurons) were validated with the same method mentioned in (PMID: 39755746)

Eukaryotic cell lines

Policy information about cell lines and Sex and Gender in Research

Cell line source(s)	ix Cells- Human Brain Microvascular Endothelial cells; 26 years old Caucasian Female; Catalog #lOHU-051; Lot #202457; 81% viability out of 0.68 million cells; NEgative for Bacteria, Yeast, Mycoplasma and other Fungi. Pathogen screen for HIV, HBV nd and HCV negative.
Authentication	Phase contrast microscopy and immunofluorescence
Mycoplasma contamination	Negative
Commonly misidentified lines (See ICLAC register)	NA

Animals and other research organisms

Policy information about studies involving animals; ARRIVE guidelines recommended for reporting animal research, and Sex and Gender in Research

Laboratory animals	C57Bl6J, B6.Cg-Tg(APPSwFILon,PSEN1*M146L*L286V)6799Vas/Mmjax and B6;C3-Tg(Prnp-MAPT*P301S)PS19Vle/J
Wild animals	NA
Reporting on sex	Eperiments were performed on male mice only as the clinical phenotypes indicate higher lmP levels in males when compared to females, and the effect sizes in males were greater than females across different measures of AD pathology. Furthermore, there was statistical significance in lowered FDG-PET uptake only in males.
Field-collected samples	NA
Ethics oversight	UW Madison IACUC- Protocol number M005599 and M006091.

Note that full information on the approval of the study protocol must also be provided in the manuscript.

Clinical data

Policy information about clinical studies

All manuscripts should comply with the ICMJE guidelines for publication of clinical research and a completed CONSORT checklist must be included with all submissions.

Clinical trial registration

Not applicable. This research is based on observational cohort studies and was not a registered clinical trial.

Study protocol

This study uses harmonized observational protocols from three University of Wisconsin-Madison studies: the Wisconsin Alzheimer's Disease Research Center (ADRC), the Wisconsin Registry for Alzheimer's Prevention (WRAP), and the Microbiome in Alzheimer's Risk Study (MARS). Data collected across these studies include neuroimaging (e.g., FDG-PET), biospecimens (blood, stool, and cerebrospinal fluid [CSF]), neuropsychological assessments (e.g., PACC3), and lifestyle and demographic information. Protocol details are available in previous publications and on study websites. Study protocols were approved by the University of Wisconsin-Madison Health Sciences Institutional Review Board under protocol ID 2013-0178 for the Wisconsin ADRC and WRAP umbrella study, and protocol ID 2015-1121 for the Microbiome in Alzheimer's Risk Study (MARS). The UW-Madison Human Research Protection Program (HRPP) explains how the campus IRBs - including the Health Sciences IRB and the Minimal Risk Research IRB - oversee all human subjects research (https://irb.wisc.edu/).

Data collection

Longitudinal clinical and cognitive data were collected by trained study staff following standardized procedures. Biospecimen collection (blood, plasma, CSF, and stool) and neuroimaging protocols were performed using validated and quality-controlled methods (https://doi.org/10.1016/j.dadm.2017.11.007). All data were collected under !RB-approved protocols with adherence to institutional and NIH guidelines for human subjects research.

Outcomes

As an observational study, no clinical intervention was performed. Primary outcomes included cognitive performance (e.g., PACC3 scores), brain glucose metabolism measured by FDG-PET, plasma and CSF biomarkers (e.g., neurofilament light chain [NfL]), and plasma and fecal biospecimens (e.g., plasma lmP and fecal microbiome). These were analyzed in relation to demographic variables. Longitudinal data were used where available to assess changes over time. See the Methods section of the manuscript for full details on outcome definitions, assessment protocols, and analytical procedures.

Plants

Seed stocks

Report on the source of all seed stocks or other plant material used. If applicable, state the seed stock centre and catalogue number. If plant specimens were collected from the field, describe the collection location, date and sampling procedures.

Novel plant genotypes

Describe the methods by which all novel plant genotypes were produced. This includes those generated by transgenic approaches, gene editing, chemical/radiation-based mutagenesis and hybridization. For transgenic lines, describe the transformation method, the number of independent lines analyzed and the generation upon which experiments were performed. For gene-edited lines, describe the editor used, the endogenous sequence targeted for editing, the targeting guide RNA sequence (if applicable) and how the editor was applied.

Authentication

Describe any authentication procedures for each seed stock used or novel genotype generated. Describe any experiments used to assess the effect of a mutation and, where applicable, how potential secondary effects (e.g. second site T-DNA insertions, mosiacism, off-target gene editing) were examined.
