# Supplementary material for: Gut bacterial metabolite imidazole propionate potentiates Alzheimer’s disease pathology
Source: Nat Commun. 2026 Jun 26;17:8017. doi: 10.1038/s41467-026-74744-z (PMC13454616; doi:10.1038/s41467-026-74744-z)
Supplement: Supplementary file 4 [file 41467_2026_74744_MOESM2_ESM.docx]

**File Name:** Supplementary Data 1

**Description:** Proteomic and phosphoproteomic level changes in mice.

**File Name:** Supplementary Data 2.

**Description:** Demographic table for cross-sectional investigation of associations between ImP and ADRD phenotypes.

**File Name:** Supplementary Data 3.

**Description:** Mediation analysis between ImP, LIBRA and PACC3 scores.

**File Name:** Supplementary Data 4.

**Description:** Longitudinal data demographics by ImP group (unique participants).

**File Name:** Supplementary Data 5.

**Description:** Demographics for Metagenomics.

**File Name:** Supplementary Data 6.

**Description:** In silico physiochemical properties of ImP.
