# Supplementary material for: Gut bacterial metabolite imidazole propionate potentiates Alzheimer’s disease pathology
Source: Nat Commun. 2026 Jun 26;17:8017. doi: 10.1038/s41467-026-74744-z (PMC13454616; doi:10.1038/s41467-026-74744-z)
Supplement: Supplementary file 5 [file 41467_2026_74744_MOESM4_ESM.docx]

**Supplementary Data 2. Demographic table for cross-sectional investigation of associations between ImP and ADRD phenotypes.**

| **Characteristic** | **N = 1,196**^1^ | **N of variables overlap with ImP** |
| --- | --- | --- |
| **ImP** |  | NA |
| Min, Max | -0.79, 1.34 |  |
| **Age (ImP)** |  | NA |
| Mean (SD), Min, Max | 61.22 (6.68), 39.43, 78.75 |  |
| **Sex** |  | 1196 |
| Female | 831 (69%) |  |
| Male | 365 (31%) |  |
| **Diagnosis** |  | 1196 |
| Normal | 1,196 (100%) |  |
| **Race** |  | 1196 |
| American Indian or Alaska Native | 14 (1.2%) |  |
| Asian | 3 (0.3%) |  |
| Black or African American | 41 (3.4%) |  |
| Other | 13 (1.1%) |  |
| White | 1,125 (94%) |  |
| **APOE e4 carrier status** |  | 1196 |
| Negative | 737 (62%) |  |
| Positive | 459 (38%) |  |
| **BMI** |  | 1196 |
| Median (Q1, Q3) | 27.78 (24.54, 32.41) |  |
| **Age (cognitive testing)** |  | 1196 |
| Median (Q1, Q3) | 61.74 (56.52, 66.25) |  |
| **PACC3 AN** |  | 958 |
| Median (Q1, Q3) | 0.09 (-0.83, 1.03) |  |
| **PACC3 CFL** |  | 1187 |
| Median (Q1, Q3) | 0.02 (-0.63, 0.61) |  |
| **PACC3 TRLB** |  | 1186 |
| Median (Q1, Q3) | 0.12 (-0.54, 0.73) |  |
| **Age difference ImP-Cog** |  | 1196 |
| Mean (SD) | 0.00 (0.00) |  |
| **Age (plasma biomarker)** |  | 1194 |
| Median (Q1, Q3) | 61.73 (56.51, 66.24) |  |
| **pTau-217** |  | 1193 |
| Median (Q1, Q3) | 0.29 (0.22, 0.40) |  |
| **Abeta 40** |  | 1194 |
| Median (Q1, Q3) | 89.30 (77.24, 104.09) |  |
| **Abeta 42** |  | 1194 |
| Median (Q1, Q3) | 6.00 (4.98, 7.08) |  |
| **GFAP** |  | 1194 |
| Median (Q1, Q3) | 92.77 (68.25, 126.35) |  |
| **NfL** |  | 1194 |
| Median (Q1, Q3) | 12.83 (9.47, 17.32) |  |
| **Abeta42/40 ratio** |  | 1194 |
| Median (Q1, Q3) | 0.07 (0.06, 0.08) |  |
| **pTau-217/Abeta42 ratio** |  | 1193 |
| Median (Q1, Q3) | 0.05 (0.04, 0.07) |  |
| **Age difference ImP-plasma biomarkers** |  | 1194 |
| Mean (SD) | 0.00 (0.00) |  |
| **Age (CSF biomarker)** |  | 224 |
| Median (Q1, Q3) | 64.34 (58.37, 68.72) |  |
| **Alpha-Synuclein** |  | 224 |
| Median (Q1, Q3) | 150.70 (116.70, 197.90) |  |
| **Abeta 1-40** |  | 222 |
| Median (Q1, Q3) | 14,310.00 (11,540.00, 17,580.00) |  |
| **Abeta 1-42** |  | 222 |
| Median (Q1, Q3) | 868.20 (622.40, 1,184.00) |  |
| **GFAP** |  | 224 |
| Median (Q1, Q3) | 8.67 (7.17, 10.97) |  |
| **IL-6** |  | 220 |
| Median (Q1, Q3) | 3.69 (3.00, 4.89) |  |
| **Neurogranin** |  | 224 |
| Median (Q1, Q3) | 792.30 (577.50, 1,022.00) |  |
| **NFL** |  | 224 |
| Median (Q1, Q3) | 83.11 (65.35, 109.90) |  |
| **pTau-181** |  | 222 |
| Median (Q1, Q3) | 17.38 (13.29, 21.58) |  |
| **S100** |  | 222 |
| Median (Q1, Q3) | 1.09 (0.92, 1.24) |  |
| **sTREM2** |  | 224 |
| Median (Q1, Q3) | 7.67 (6.29, 9.74) |  |
| **tTau** |  | 222 |
| Median (Q1, Q3) | 199.80 (156.00, 246.50) |  |
| **YKL-40** |  | 224 |
| Median (Q1, Q3) | 142.70 (120.00, 178.70) |  |
| **Abeta 1-42/1-40 derived** |  | 222 |
| Median (Q1, Q3) | 0.07 (0.05, 0.08) |  |
| **pTau/Abeta 1-42 derived** |  | 222 |
| Median (Q1, Q3) | 0.02 (0.01, 0.03) |  |
| **tTau/Abeta 1-42 derived** |  | 222 |
| Median (Q1, Q3) | 0.21 (0.17, 0.31) |  |
| **Inverse Abeta 1-42 derived** |  | 222 |
| Median (Q1, Q3) | 0.001 (0.0008, 0.0017) |  |
| **Age difference ImP-CSF biomarkers** |  | 224 |
| Mean (SD) | 0.83 (1.09) |  |
| **Age (FDG PET)** |  | 168 |
| Median (Q1, Q3) | 63.36 (58.50, 66.95) |  |
| **SUVR all regions (avg)** |  | 168 |
| Median (Q1, Q3) | 0.98 (0.96, 1.02) |  |
| **Frontal SUVR (avg)** |  | 168 |
| Median (Q1, Q3) | 1.04 (0.99, 1.09) |  |
| **Parietal SUVR (avg)** |  | 168 |
| Median (Q1, Q3) | 1.04 (0.99, 1.08) |  |
| **Temporal SUVR (avg)** |  | 168 |
| Median (Q1, Q3) | 0.94 (0.90, 0.96) |  |
| **Occipital SUVR (avg)** |  | 168 |
| Median (Q1, Q3) | 1.07 (1.02, 1.11) |  |
| **Age difference ImP-FDG** |  | 168 |
| Mean (SD) | 0.43 (0.62) |  |
| **Age (LIBRA assessment)** |  | 1196 |
| Median (Q1, Q3) | 61.74 (56.52, 66.25) |  |
| **LIBRA Score** |  | 1194 |
| Median (Q1, Q3) | 0.60 (-1.00, 2.20) |  |
| **Age difference ImP-LIBRA** |  | 1196 |
| Mean (SD) | 0.00 (0.00) |  |
| ^1^n (%) | |  |
