# Supplementary material for: Gut bacterial metabolite imidazole propionate potentiates Alzheimer’s disease pathology
Source: Nat Commun. 2026 Jun 26;17:8017. doi: 10.1038/s41467-026-74744-z (PMC13454616; doi:10.1038/s41467-026-74744-z)
Supplement: Supplementary file 6 [file 41467_2026_74744_MOESM5_ESM.docx]

**Supplementary Data 3. Mediation analysis between ImP, LIBRA and PACC3 scores.**

| **outcome** | **path** | **coef** | **CI[2.5%]** | **CI[97.5%]** | **se** | **pval** | **pval_fdr** | **significant_fdr** | **prop_mediated (indirect/total)** |
| --- | --- | --- | --- | --- | --- | --- | --- | --- | --- |
| pacc3_an_xw_z | LIBRA_Score ~ X | 0.630385 | 0.179592 | 1.081177 | 0.229707 | 0.006178 |  | FALSE |  |
| pacc3_an_xw_z | Y ~ LIBRA_Score | -0.10943 | -0.14886 | -0.07 | 0.020091 | 6.53E-08 |  | FALSE |  |
| pacc3_an_xw_z | Total | -0.41208 | -0.69532 | -0.12885 | 0.144327 | 0.004395 |  | FALSE |  |
| pacc3_an_xw_z | Direct | -0.34582 | -0.62632 | -0.06532 | 0.142932 | 0.01573 |  | FALSE |  |
| pacc3_an_xw_z | Indirect | -0.06626 | -0.13648 | -0.01273 | 0.031191 | 0.0216 | 0.0216 | TRUE | 0.160796 |
| pacc3_cfl_xw_z | LIBRA_Score ~ X | 0.602663 | 0.192187 | 1.013138 | 0.209215 | 0.004041 |  | FALSE |  |
| pacc3_cfl_xw_z | Y ~ LIBRA_Score | -0.07281 | -0.09965 | -0.04598 | 0.013678 | 1.22E-07 |  | FALSE |  |
| pacc3_cfl_xw_z | Total | -0.3195 | -0.51433 | -0.12467 | 0.099304 | 0.001329 |  | FALSE |  |
| pacc3_cfl_xw_z | Direct | -0.27756 | -0.47105 | -0.08408 | 0.098618 | 0.004966 |  | FALSE |  |
| pacc3_cfl_xw_z | Indirect | -0.04194 | -0.08583 | -0.01071 | 0.018916 | 0.0128 | 0.0192 | TRUE | 0.131264 |
| pacc3_trlb_xw_z | LIBRA_Score ~ X | 0.661532 | 0.248413 | 1.07465 | 0.210562 | 0.001721 |  | FALSE |  |
| pacc3_trlb_xw_z | Y ~ LIBRA_Score | -0.08512 | -0.1097 | -0.06055 | 0.012527 | 1.71E-11 |  | FALSE |  |
| pacc3_trlb_xw_z | Total | -0.34601 | -0.52668 | -0.16534 | 0.092084 | 0.00018 |  | FALSE |  |
| pacc3_trlb_xw_z | Direct | -0.29213 | -0.47045 | -0.1138 | 0.09089 | 0.001344 |  | FALSE |  |
| pacc3_trlb_xw_z | Indirect | -0.05388 | -0.10458 | -0.01585 | 0.022007 | 0.006 | 0.018 | TRUE | 0.155725 |
