# Supplementary material for: Gut bacterial metabolite imidazole propionate potentiates Alzheimer’s disease pathology
Source: Nat Commun. 2026 Jun 26;17:8017. doi: 10.1038/s41467-026-74744-z (PMC13454616; doi:10.1038/s41467-026-74744-z)
Supplement: Supplementary file 7 [file 41467_2026_74744_MOESM6_ESM.docx]

Supplementary Data 4. Longitudinal data demographics by ImP group (unique participants).

|  | **High** | | | **Low** | | |
| --- | --- | --- | --- | --- | --- | --- |
|  | **Visit1**  N = 223^1^ | **Visit2**  N = 222^1^ | **Visit3**  N = 46^1^ | **Visit1**  N = 242^1^ | **Visit2**  N = 242^1^ | **Visit3**  N = 61^1^ |
| **ImP (average across visits)** |  |  |  |  |  |  |
| Mean (SD) | 0.37 (0.29) | 0.38 (0.26) | 0.32 (0.22) | -0.23 (0.12) | -0.23 (0.13) | -0.24 (0.14) |
| **Age (ImP sample)** |  |  |  |  |  |  |
| Mean (SD) | 62 (6) | 65 (6) | 65 (6) | 60 (7) | 62 (7) | 63 (7) |
| **Age (Cognitive assessment)** |  |  |  |  |  |  |
| Mean (SD) | 64 (6) | 65 (6) | 64 (6) | 61 (7) | 62 (6) | 62 (7) |
| **Age difference (ImP - Cognition)** |  |  |  |  |  |  |
| Mean (SD) | 2.97 (0.66) | 3.07 (0.81) | 3.26 (0.53) | 3.05 (0.60) | 3.19 (0.72) | 3.33 (0.57) |
| **Age (Plasma biomarker)** |  |  |  |  |  |  |
| Mean (SD) | 62 (6) | 65 (6) | 65 (6) | 60 (7) | 62 (7) | 63 (7) |
| **Age difference (ImP - Biomarker)** |  |  |  |  |  |  |
| Mean (SD) | 0.0001 (0.0013) | 0.0108 (0.1597) | 0.0004 (0.0021) | 0.0002 (0.0026) | 0.0002 (0.0029) | 0.0008 (0.0028) |
| **Sex** |  |  |  |  |  |  |
| Female | 115 (52%) | 115 (52%) | 22 (48%) | 220 (91%) | 220 (91%) | 54 (89%) |
| Male | 108 (48%) | 107 (48%) | 24 (52%) | 22 (9.1%) | 22 (9.1%) | 7 (11%) |
| **Race** |  |  |  |  |  |  |
| American Indian or Alaska Native | 3 (1.3%) | 3 (1.4%) | 0 (0%) | 1 (0.4%) | 1 (0.4%) | 0 (0%) |
| Asian | 2 (0.9%) | 2 (0.9%) | 0 (0%) | 0 (0%) | 0 (0%) | 0 (0%) |
| Black or African American | 7 (3.1%) | 7 (3.2%) | 0 (0%) | 6 (2.5%) | 6 (2.5%) | 0 (0%) |
| Other | 2 (0.9%) | 2 (0.9%) | 0 (0%) | 1 (0.4%) | 1 (0.4%) | 0 (0%) |
| White | 209 (94%) | 208 (94%) | 46 (100%) | 234 (97%) | 234 (97%) | 61 (100%) |
| **APOE e4 carrier status** |  |  |  |  |  |  |
| Negative | 138 (62%) | 137 (62%) | 28 (61%) | 150 (62%) | 150 (62%) | 37 (61%) |
| Positive | 85 (38%) | 85 (38%) | 18 (39%) | 92 (38%) | 92 (38%) | 24 (39%) |
| **BMI** |  |  |  |  |  |  |
| Mean (SD) | 31 (7) | 31 (7) | 31 (5) | 27 (6) | 28 (6) | 27 (5) |
| **Number of repeated cognitive tests** |  |  |  |  |  |  |
| Mean (SD) | 2.65 (0.49) | 2.97 (0.68) | 3.18 (0.50) | 2.76 (0.45) | 3.12 (0.63) | 3.24 (0.62) |
| **PACC3 Animal Naming** |  |  |  |  |  |  |
| Mean (SD) | -0.16 (1.22) | -0.22 (1.22) | -0.05 (1.23) | 0.46 (1.14) | 0.42 (1.15) | 0.78 (1.04) |
| **PACC3 Letter Fluency** |  |  |  |  |  |  |
| Mean (SD) | -0.19 (0.87) | -0.23 (0.87) | -0.18 (0.86) | 0.30 (0.84) | 0.28 (0.84) | 0.43 (0.76) |
| **PACC3 Trail Making Test-B** |  |  |  |  |  |  |
| Mean (SD) | -0.13 (0.81) | -0.16 (0.82) | -0.05 (0.79) | 0.38 (0.76) | 0.36 (0.78) | 0.61 (0.65) |
| **Log10 pTau217** |  |  |  |  |  |  |
| Mean (SD) | -0.48 (0.21) | -0.46 (0.23) | -0.49 (0.22) | -0.53 (0.22) | -0.51 (0.26) | -0.50 (0.21) |
| **Log10 NfL** |  |  |  |  |  |  |
| Mean (SD) | 1.13 (0.21) | 1.18 (0.21) | 1.17 (0.17) | 1.11 (0.21) | 1.14 (0.19) | 1.17 (0.18) |
| ^1^n (%) | | | | | | |
