# Supplementary material for: Gut bacterial metabolite imidazole propionate potentiates Alzheimer’s disease pathology
Source: Nat Commun. 2026 Jun 26;17:8017. doi: 10.1038/s41467-026-74744-z (PMC13454616; doi:10.1038/s41467-026-74744-z)
Supplement: Supplementary file 8 [file 41467_2026_74744_MOESM7_ESM.docx]

**Supplementary Data 5. Demographics for Metagenomics.**

| **Characteristic** | **N = 294**^1^ | **N of variables overlap with MAGs** |
| --- | --- | --- |
| **Unique participants with MAGs** |  | NA |
| N | 294 (100%) |  |
| **Age (fecal sample)** |  | 294 |
| Mean (SD), Min, Max | 67.58 (7.13), 47.49, 90.49 |  |
| **Sex** |  | 294 |
| Female | 190 (65%) |  |
| Male | 104 (35%) |  |
| **Race** |  | 294 |
| American Indian or Alaska Native | 1 (0.3%) |  |
| Asian | 1 (0.3%) |  |
| Black or African American | 32 (11%) |  |
| Other | 1 (0.3%) |  |
| White | 259 (88%) |  |
| **Diagnosis** |  | 294 |
| Dementia-AD | 22 (7.5%) |  |
| Impaired-Other | 3 (1.0%) |  |
| MCI-AD | 11 (3.7%) |  |
| MCI-Other | 2 (0.7%) |  |
| Normal | 256 (87%) |  |
| **APOE e4 carrier status** |  | 294 |
| Negative | 178 (61%) |  |
| Positive | 116 (39%) |  |
| **BMI** |  | 294 |
| Median (Q1, Q3) | 28.05 (25.03, 31.88) |  |
| **Age (cognitive testing)** |  | 274 |
| Median (Q1, Q3) | 66.90 (62.73, 71.62) |  |
| **PACC3 AN** |  | 274 |
| Median (Q1, Q3) | -0.14 (-1.70, 1.00) |  |
| **PACC3 CFL** |  | 273 |
| Median (Q1, Q3) | -0.18 (-1.10, 0.73) |  |
| **PACC3 TRLB** |  | 249 |
| Median (Q1, Q3) | -0.16 (-1.04, 0.72) |  |
| **Age difference ImP-Cog** |  | 274 |
| Median (Q1, Q3) | 0.46 (0.21, 0.83) |  |
| **Age (plasma biomarker)** |  | 242 |
| Median (Q1, Q3) | 66.92 (63.11, 71.26) |  |
| **pTau-217** |  | 234 |
| Median (Q1, Q3) | 0.30 (0.22, 0.47) |  |
| **Abeta 40** |  | 242 |
| Median (Q1, Q3) | 100.47 (89.15, 115.17) |  |
| **Abeta 42** |  | 242 |
| Median (Q1, Q3) | 6.61 (5.43, 7.85) |  |
| **GFAP** |  | 242 |
| Median (Q1, Q3) | 116.79 (82.22, 157.04) |  |
| **NfL** |  | 242 |
| Median (Q1, Q3) | 15.31 (11.96, 20.81) |  |
| **Abeta42/40 ratio** |  | 242 |
| Median (Q1, Q3) | 0.07 (0.06, 0.07) |  |
| **pTau-217/Abeta42 ratio** |  | 234 |
| Median (Q1, Q3) | 0.04 (0.03, 0.08) |  |
| **Age difference ImP-plasma biomarkers** |  | 242 |
| Median (Q1, Q3) | 0.59 (0.21, 1.19) |  |
| **Age (CSF biomarker)** |  | 113 |
| Median (Q1, Q3) | 66.50 (61.77, 71.03) |  |
| **Alpha-Synuclein** |  | 113 |
| Median (Q1, Q3) | 156.10 (110.70, 192.10) |  |
| **Abeta 1-40** |  | 112 |
| Median (Q1, Q3) | 14,285.00 (11,162.50, 17,125.00) |  |
| **Abeta 1-42** |  | 112 |
| Median (Q1, Q3) | 738.70 (561.28, 1,089.75) |  |
| **GFAP** |  | 113 |
| Median (Q1, Q3) | 9.13 (7.93, 12.14) |  |
| **IL-6** |  | 112 |
| Median (Q1, Q3) | 3.66 (2.98, 4.84) |  |
| **Neurogranin** |  | 113 |
| Median (Q1, Q3) | 758.80 (559.10, 1,011.00) |  |
| **NFL** |  | 113 |
| Median (Q1, Q3) | 90.83 (70.49, 121.40) |  |
| **pTau-181** |  | 112 |
| Median (Q1, Q3) | 18.04 (13.58, 23.89) |  |
| **S100** |  | 112 |
| Median (Q1, Q3) | 1.13 (0.99, 1.29) |  |
| **sTREM2** |  | 113 |
| Median (Q1, Q3) | 7.88 (6.40, 9.85) |  |
| **tTau** |  | 112 |
| Median (Q1, Q3) | 208.60 (158.08, 259.88) |  |
| **YKL-40** |  | 113 |
| Median (Q1, Q3) | 142.60 (121.90, 192.70) |  |
| **Abeta 1-42/1-40 derived** |  | 112 |
| Median (Q1, Q3) | 0.06 (0.04, 0.07) |  |
| **pTau/Abeta 1-42 derived** |  | 112 |
| Median (Q1, Q3) | 0.02 (0.02, 0.03) |  |
| **tTau/Abeta 1-42 derived** |  | 112 |
| Median (Q1, Q3) | 0.24 (0.19, 0.38) |  |
| **Inverse Abeta 1-42 derived** |  | 112 |
| Median (Q1, Q3) | 0.001 (0.0009, 0.002) |  |
| **Age difference ImP-CSF biomarkers** |  | 113 |
| Median (Q1, Q3) | 0.96 (0.55, 1.67) |  |
| ^1^n (%) | |  |
