# Supplementary material for: Gut bacterial metabolite imidazole propionate potentiates Alzheimer’s disease pathology
Source: Nat Commun. 2026 Jun 26;17:8017. doi: 10.1038/s41467-026-74744-z (PMC13454616; doi:10.1038/s41467-026-74744-z)
Supplement: Supplementary file 9 [file 41467_2026_74744_MOESM8_ESM.docx]

**Supplementary Data 6. *In silico* physiochemical properties of ImP.**

| **Molecule** | **Imidazole Propionate** |
| --- | --- |
| Canonical SMILES | OC(=O)CCc1cnc[nH]1 |
| Formula | C6H8N2O2 |
| MW | 140.14 |
| #Heavy atoms | 10 |
| #Aromatic heavy atoms | 5 |
| Fraction Csp3 | 0.33 |
| #Rotatable bonds | 3 |
| #H-bond acceptors | 3 |
| #H-bond donors | 2 |
| MR | 34.94 |
| TPSA | 65.98 |
| iLOGP | 0.28 |
| XLOGP3 | -0.15 |
| WLOGP | 0.43 |
| MLOGP | -0.69 |
| Silicos-IT Log P | 0.93 |
| Consensus Log P | 0.16 |
| ESOL Log S | -0.79 |
| ESOL Solubility (mg/ml) | 2.29E+01 |
| ESOL Solubility (mol/l) | 1.64E-01 |
| ESOL Class | Very soluble |
| Ali Log S | -0.78 |
| Ali Solubility (mg/ml) | 2.32E+01 |
| Ali Solubility (mol/l) | 1.66E-01 |
| Ali Class | Very soluble |
| Silicos-IT LogSw | -1.39 |
| Silicos-IT Solubility (mg/ml) | 5.70E+00 |
| Silicos-IT Solubility (mol/l) | 4.06E-02 |
| Silicos-IT class | Soluble |
| GI absorption | High |
| BBB permeant | No |
| Pgp substrate | No |
| CYP1A2 inhibitor | No |
| CYP2C19 inhibitor | No |
| CYP2C9 inhibitor | No |
| CYP2D6 inhibitor | No |
| CYP3A4 inhibitor | No |
| log Kp (cm/s) | -7.26 |
| Lipinski #violations | 0 |
| Ghose #violations | 3 |
| Veber #violations | 0 |
| Egan #violations | 0 |
| Muegge #violations | 1 |
| Bioavailability Score | 0.55 |
| PAINS #alerts | 0 |
| Brenk #alerts | 0 |
| Leadlikeness #violations | 1 |
| Synthetic Accessibility | 1.73 |
| Brenk #alerts | 0 |
| Leadlikeness #violations | 1 |
| Synthetic Accessibility | 1.73 |
